# Novel Polyurethanes Based on Recycled Polyethylene Terephthalate: Synthesis, Characterization, and Formulation of Binders for Environmentally Responsible Rocket Propellants

**DOI:** 10.3390/polym13213828

**Published:** 2021-11-05

**Authors:** Florin Marian Dîrloman, Gabriela Toader, Traian Rotariu, Tudor Viorel Țigănescu, Raluca Elena Ginghină, Răzvan Petre, Florentina Alexe, Mihai Ionuț Ungureanu, Edina Rusen, Aurel Diacon, Adi Ghebaur, Monica Duldner, Alina Elena Coman, Robert Țincu

**Affiliations:** 1 Military Technical Academy “Ferdinand I”, 39-49 George Cosbuc Boulevard, 050141 Bucharest, Romania; florin.dirloman@mta.ro (F.M.D.); gabriela.toader@mta.ro (G.T.); viorel.tiganescu@mta.ro (T.V.Ț.); mihai.ungureanu@mta.ro (M.I.U.); 2 Military Equipment and Technologies Research Agency, 16 Aeroportului Street, Clinceni, 077025 Ilfov, Romania; 3 Research and Innovation Center for CBRN Defense and Ecology, 225 Soseaua Oltenitei, 041327 Bucharest, Romania; raluca.ginghina@nbce.ro (R.E.G.); razvan.petre@nbce.ro (R.P.); florentina.alexe@nbce.ro (F.A.); 4 Faculty of Applied Chemistry and Materials Science, University Politehnica of Bucharest, 1-7 Gh. Polizu Street, 011061 Bucharest, Romania; edina.rusen@upb.ro (E.R.); aurel.diacon@upb.ro (A.D.); adi.ghebaur@upb.ro (A.G.); 5 National Institute of Research and Development for Chemistry and Petrochemistry, 202 Splaiul Independentei, 060041 Bucharest, Romania; monica.duldner@icechim.ro (M.D.); alina-elena.coman@icechim.ro (A.E.C.); 6 Center for Organic Chemistry “C.D. Nenitescu” of the Romanian Academy, 202B Splaiul Independentei, 060023 Bucharest, Romania; tincurobert@gmail.com

**Keywords:** polyethylene terephthalate recycling, polyurethane, binder, polymeric composite, rocket propellant, mechanical properties, subscale rocket motor

## Abstract

Novel polyurethane-based binders, specifically designed for environmentally responsible rocket propellant composites, were obtained by employing the polyester-polyols that resulted from the degradation of polyethylene terephthalate waste. A new class of “greener” rocket propellants, comprising polyurethanes (based on recycled PET) as the binder, phase stabilized ammonium nitrate (PSAN) as the eco-friendly oxidizer, and triethylene glycol dinitrate (TEGDN) as the energetic plasticizer, together with aluminum as fuel and Fe_2_O_3_ as the catalyst, is herein reported. The components of the energetic mixtures were investigated (individually and as composite materials) through specific analytical tools: ^1^H-NMR, FT-IR, SEM-EDX, DTA and TGA, tensile and compression tests, DMA, and micro-CT. Moreover, the feasibility of this innovative solution is sustained by the ballistic performances exhibited by these composite materials in a subscale rocket motor, proving that these new formulations are suitable for rocket propellant applications.

## 1. Introduction

Solid rocket propellants are a particular class of energetic materials that are developed to ensure the propulsion of spacecrafts, missiles, and rockets toward a target. The main energetic transformation is represented by steady combustion in an enclosed environment, specifically designed to release hot gases at high speeds to obtain the appropriate propulsion thrust [1]. The main aspects that influence the combustion behavior are the constituent elements and the configuration of the grains. Structurally, solid rocket composite propellants are typically based on a heterogeneous combination of distinct compounds that serve as fuel, oxidizer, burn-rate modifiers, and binder [1,2]. Usually, the applicability of polyurethane or polyurea matrices lies in the field of thermal insulations, hydro-isolations, adhesives, and ballistic protection [3,4,5,6]. In the case of energetic materials, they have a dual purpose, as a binding agent and as an organic fuel [1,2]. These polymeric matrices are used for binding other solid components of the rocket propellants, to protect them against environmental agents and to confer a certain geometry and mechanical strength. In state-of-the-art composite rocket propellants, ammonium perchlorate (AP) is the preferred oxidizer, almost without exception, due to its outstanding properties [1,2,7,8]. However, in the last decade, environmental impact issues have urged research activities for a replacement with “green” oxidizers, such as phase-stabilized ammonium nitrate (PSAN) or ammonium dinitramide (ADN) [2,9,10,11]. A common practice in rocket propellant technology consists in adding metallic additives to increase the heat output, the density, and the specific impulse of the energetic material. Aluminum fine powder, magnesium, and boron are the main candidates as fuels in rocket propellant formulations. Together with the oxidizers and additives, the solid fuel is introduced in the polymeric matrix to ensure homogeneity, protection, and proper mechanical characteristics of the energetic mixture [2,8,12,13,14,15,16].

Historically, the beginning of the use of polymers in the development of solid rocket propellants is marked by the introduction of nitrocellulose (NC). NC was mainly employed in homogeneous (colloidal) rocket propellants, providing the necessary structural integrity to the propellants so that they could be molded into different geometric configurations, according to the type of launching system [1,2,17]. In heterogeneous propellants, polysulfide polymers were initially used as the binding agent. The drawback of this polymer was the high incompatibility with metallic fuels, causing safety problems occasionally leading to autoignition during long-term storage [2,18]. In this context, the necessity of developing a new class of binders became imperative. The polybutadiene chain was found to be a more suitable binder for the energetic composites, due to its high elasticity and low glass temperature. The copolymer of butadiene and acrylic acid (PBAA) was the first one to be used from this class [1,2,18,19]. The low viscosity of these binders allowed significant solid loading ratios of oxidizer and metallic fuel in the energetic composites. However, these materials exhibited poor mechanical properties. This aspect was overcome using a polybutadiene acrylonitrile copolymer (PBAN). The low cost of production, the low viscosity, and the impulse increase made PBAN, at that time, the ideal candidate as a rocket propellant binder [1,2,18,19,20,21]. However, the high temperature required to cure these propellants led to the development of other types of binders, such as carboxyl-terminated polybutadiene (CTPB). CTPB-based composites displayed significantly enhanced mechanical properties, especially at lower temperatures, in comparison with PBAA or PBAN binders, without affecting the specific impulse, density, or solid loading ratios [1,2,18,19]. The curative agents used for these types of polymers employed as binders are usually based on di- or tri-functional epoxides or aziridines [18]. Modern solid rocket propellants use hydroxyl-terminated polybutadiene (HTPB), an inert binder from the same class as CTPB, which ensures the optimal combination of thermodynamic and mechanical properties [7,8,18,20]. The HTPB binders exhibit superior elongation capacity at low temperatures and better ageing properties in contrast with CTPB [18,19]. The curing process of HTPB is quicker due to the use of isocyanates [19,22,23]. Despite the advantages of HTPB binders, the problem of using this type of inert binder in a composite rocket propellant is represented by the necessity of adding an extra amount of oxidizer to balance the oxygen score and achieve the appropriate performances. Consequently, it is desirable to develop more versatile polymers with substantial energetic character. The integration of azido- or nitro-functional groups on the polymeric chain is conducted for a significant performance enhancement with lower amounts of energetic materials. Among the polymers employed as energetic binders in solid rocket propellants, two worth mentioning are polyglycidyl nitrates (PGN) and glycidyl azido polymers (GAP) [10,18]. GAP are considered excellent energetic polymers due to their unique structure, which ensures a high heat of formation. These polymers are recommended for rocket propellant composites that comprise eco-friendly oxidizers with weaker energetic characteristics, such as ammonium nitrate, to balance the energy loss caused by replacing classical oxidizers such as ammonium perchlorate [10]. 

In recent times, the pollution of ecosystems due to the inappropriate disposal of PET waste is widespread. Moreover, the high level of carbon dioxide emissions released into the air because of their combustion has led to the application of preventive measures. As a countermeasure to these ecological issues, interest in the development of materials based on recycled PET has achieved substantial growth [24]. As a result, in December 2015, the European Commission adopted a plan based on a circular economy strategy that will be applied in the context of reusing plastic wastes [24,25,26,27]. For obtaining eco-friendly polyols, with extensive applicability, PET waste can be recycled by undergoing a controlled degradation procedure that aims to generate oligomers that can be subsequently utilized for specific applications [27,28,29,30]. 

In this paper, a new group of flexible polyurethanes was synthesized from polyols obtained through the recycling of PET waste. A commercial polyol and a commercial aromatic polyisocyanate were also employed for the development of polyurethane formulations. These polyurethanes were subsequently used as binders in various rocket propellant composites, also comprising an eco-friendly oxidizer. The polyurethane networks were softened with an energetic plasticizer (triethylene glycol dinitrate, TEGDN) to boost the exothermic decomposition of these rocket propellants and to obtain composites with appropriate mechanical behavior suitable for this type of application. The synthesized eco-friendly composites can be successfully employed as rocket propellants, an aspect that was demonstrated through the subscale rocket motor experimental investigations that we performed.

Therefore, the novelty of this work consists in the innovative path of demonstrating the applicability of novel synthesized polyurethanes as binders for new environmentally friendly rocket propellants, herein reported. These original energetic mixtures, comprising newly synthesized components such as polyurethanes (from recycled PET) as the binder, PSAN as the eco-friendly oxidizer, TEGDN as the energetic plasticizer, together with aluminum for fuel and Fe_2_O_3_ as the catalyst, achieved remarkable performances as rocket propellants, demonstrable by the results obtained through the measurements performed using the subscale rocket motor. 

Furthermore, the experimental data revealed that these new “green” binders could successfully replace classical binders from existing rocket propellants, since they possess comparable performances (similar thermal stability, up to 300 °C [31], similar mechanical properties [32]), but multiple advantages in comparison with HTPB binders [33], emphasized in the experimental section. The new rocket propellant composites, besides their environmentally friendly character, displayed better performances: up to a 30% improvement of the specific volume (in comparison with HTPB-based rocked propellant from consecrated aviation missiles). The heat of combustion (≈1000 cal/g) and T_g_ values (−60 °C to −32 °C) were comparable with nitrocellulose double base propellants (≈1020 cal/g and −35.5 °C) [34,35], which ensure that they maintain their performances even at lower environmental temperatures. The polyols obtained from PET degradation, the polyurethanes, and the composite rocket propellant formulations were investigated through specific analytical tools. Moreover, the feasibility of these novel energetic composites was demonstrated through the evaluation of the combustion rate and maximum pressure, proving that these new materials are suitable for rocket propellant applications. 

Polyurethane networks, mainly those based on HTPB, are currently considered state-of-the-art propellant binder systems, being extensively used for composite solid propellants. Unfortunately, as already described above, they possess several disadvantages, among which the drawbacks of their inadequate life-cycle assessment can also be mentioned. In contrast, the materials developed in this study are especially designed to be environmentally friendly, while ensuring improved performances as new “green” alternatives for rocket propellants.

## 2. Materials and Methods

### 2.1. Materials

#### 2.1.1. Materials for the Catalytic Degradation of Polyethylene Terephthalate (PET)

PET (Mn ≈ 40,000–70,000 Da, melting range: 254–260 °C) was obtained from post-consumer bottles cut into square shaped flakes (5 mm × 5 mm). The PET flakes were washed with distilled water and dried at 100 °C for 6 h before being introduced into the degradation reactor. Poly(ethylene glycol) of an average Mn≈ 570–630 Da (PEG600, Sigma Aldrich, St. Louis, MO, USA), titanium(IV) isopropoxide (TTIP, 97%, Sigma Aldrich, St. Louis, MO, USA), adipic acid (AA, 99%, Sigma Aldrich, St. Louis, MO, USA), sebacic acid (SA, 99%, Sigma Aldrich, St. Louis, MO, USA), and tin(II) 2-ethylhexanoate (C_16_H_30_O_4_Sn, 92.5–100%, Sigma Aldrich, St. Louis, MO, USA) were used as received.

#### 2.1.2. Materials for Polyurethane Synthesis

The two types of polyester-polyols (named RP1 and RP2 in this paper, according to the compositions described below in ‘Methods’ section) that resulted from PET degradation were vacuum dried for 24 h at 50 °C and were subsequently used for polyurethane synthesis. Setathane D1160, hydroxyl content 5.4%, (SET, Allnex, Brussels, Belgium) was vacuum dried for 24 h at 50 °C before being employed in polyurethane synthesis. Diphenylmethane–4,4′–diisocyanate, -NCO content 31.5% (MDI, technical product Desmodur^®^ 44V20L, Covestro, Leverkusen, Germany), was used as received. The energetic plasticizer, triethylene glycol dinitrate (TEGDN), was synthesized and purified in the Military Technical Academy (MTA) according to a procedure found in the literature [36].

#### 2.1.3. Materials for Composite Rocket Propellants Based on Polyurethane Binders

The above-mentioned synthesized polyurethanes were employed as binder for the new rocket propellant composite formulations. The “green” oxidizer, phase-stabilized ammonium nitrate (PSAN), was prepared in MTA: ammonium nitrate (AN, min. 98%, Honeywell Fluka™, Seelze, Germany) and potassium nitrate (KN, 99%, ACROS Organics™, Fair Lawn, NJ, USA) were co-crystallized from aqueous solution, according to the procedure described in the literature [37]. As metallic fuel, two types of aluminum powder, with an average particle size <5 μm (99.5%, Sigma Aldrich, St. Louis, MO, USA) and 100 μm (99.5%, Sigma Aldrich, St. Louis, MO, USA), were used as received. As catalyst, iron oxide (99.9%, powder, Fe_2_O_3_, Sigma Aldrich, St. Louis, MO, USA) was employed as received.

### 2.2. Methods

#### 2.2.1. Polyester-Polyols Synthesis through the Catalytic Degradation of Polyethylene Terephthalate (PET)

**Scheme 1:**
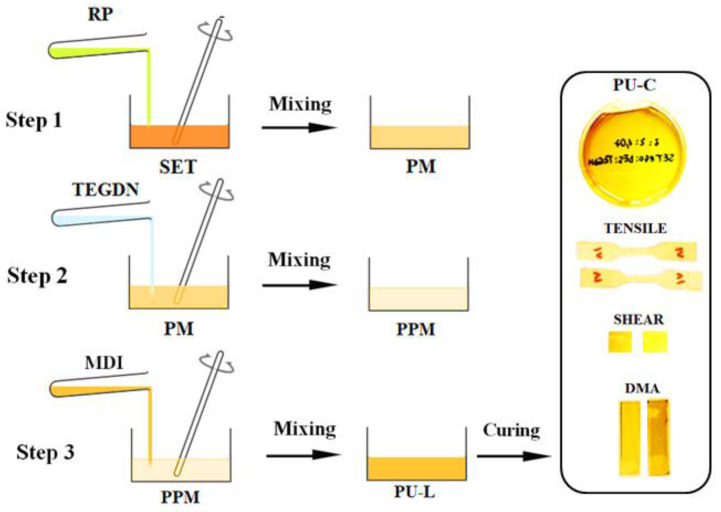
Schematic presentation of the synthesis and curing of the polyurethane binders (PM: polyols mixture; PPM: plasticizer-polyols mixture; PU-L: polyurethane liquid state; PU-C: polyurethane cured state).

The polyester-polyols (RP1 and RP2) necessary for the synthesis of the binder were obtained following two main steps. In the first step, 1 mol of PET, 1.8 mol of PEG600, and tin(II) 2-ethylhexanoate (0.5% from PET wt.) were introduced in a four-neck round-bottom flask (with inlets fitted for a mechanical stirrer, a thermometer, a reflux condenser, and a nitrogen gas purging line), and they were maintained under an inert atmosphere, at total reflux, with continuous stirring, for 3 h at 190 °C. The reaction mixture was then cooled below 100 °C. In the second step were added 0.3 mol of PEG600, 0.5 mol of AS, 0.5 mol of AA, and TIPT (0.5% from PET wt.), and the reaction mixture was maintained under inert atmosphere, at total reflux, with continuous stirring, for another 4–6 h at 190–200 °C. The main possible reaction products obtained through this two-step catalytic degradation are illustrated in Scheme 1. RP1 and RP2 were obtained according to the procedure described, with the following observations: RP1 was obtained by employing both AA and SA (0.5 mol of AS, 0.5 mol of AA), while RP2 was obtained by employing only AA (0.75 mol of AA).

#### 2.2.2. Synthesis of Polyurethane Binder

Starting from the reaction products described, a series of miscible blends was prepared by mixing (Scheme 1, Step 1 and Step 2) the newly synthesized polyester-polyols (RP1 or RP2) with the commercial polyol (SET) and with the synthesized energetic plasticizer (TEGDN). Therefore, RP1, RP2, SET, and TEGDN were mixed in different molar ratios, as detailed in Table 1 (Scheme 1, Step 1 and Step 2), before polyurethanes synthesis. 

To investigate the possibility of utilizing these newly obtained polyurethanes as binders in rocket propellant composites, polyurethane formulations were obtained by following two working strategies: the first one employing only a commercial polyester-polyol (SET) and the second one consisted in the addition of the polyester-polyols that resulted from the catalytic degradation of PET (RP1 and RP2) to the commercial polyol (SET), as described in Table 1. Thus, the polyurethanes were obtained by reacting the commercial polyol (SET) and the recycled polyols (RP1 and RP2) with the curing agent Desmodur^®^ 44V20L (MDI, diphenylmethane-4,4′-diisocyanate) by adjusting the -NCO/-OH molar ratios, according to the compositions detailed in Table 2. The energetic plasticizer, TEGDN, was added in two different proportions: 15 wt.% and 30 wt.% of the total binder content, according to Table 1 and Table 2. The polyurethane synthesis process occurred in one simple step: MDI was added to the polyol blends (Table 1 and Table 2) and the mixture was stirred vigorously (except for binders that also included energetic plasticizer (TEGDN), which was introduced into the polyol blends before the addition of the curing agent). The mixing process was carried out at 50 °C to improve processability. The resulting compounds were poured into glass molds, and they were allowed to cure at 60 °C in a vacuum oven. The polyurethane films were exfoliated from the glass molds after the completion of the curing process and were subjected to specific investigations, as described in Results section. 

#### 2.2.3. Rocket Propellant Composite Formulations

**Scheme 2:**
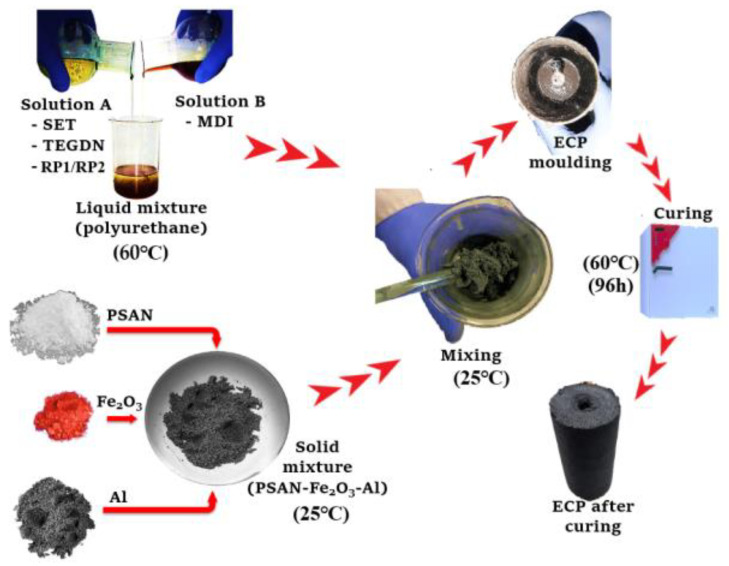
Schematic representation of the rocket propellant composite fabrication process.

The new composite formulations consisted in a solid–liquid mixture, in which the liquid component, represented by the novel polyurethane binders, incorporated the solid components, comprising an oxidizer (PSAN, two granulometric sizes), a metallic fuel (two granulometric sizes of aluminum powder), and catalyst (iron oxide). To provide adequate mechanical strength for these rocket propellant composites and to facilitate the mixing process, the oxidizer chosen for this application used bi-granular (200 μm and 50 μm) mixture. To ensure a good homogeneity of the composite rocket propellant, the liquid and the solid phases were mixed separately, as follows: the liquid mixture was prepared by simply mixing the polyols with the curing agent (MDI); separately, the solid mixture containing the oxidant (PSAN), the metallic fuel (Al), and the catalyst (Fe_2_O_3_) was sieved and sorted by granule size, then dried. Then, the liquid and the solid phases were thoroughly mixed and introduced in cylindrical molds with 45 mm diameter and allowed to cure at 60 °C in a vacuum oven. The described procedure can be better understood in conjunction with the illustrations presented in Scheme 2. 

The novel composite rocket propellant formulations were produced in small batches (25 g) and pressed at 10 bar (as required by the characteristic tests necessary for the evaluation of the performances of solid rocket propellants: evaluation of combustion behavior, evaluation of the mechanical properties, and morphological analysis) [38]. Subsequently, the samples were cured under vacuum for 96 h at 60 °C. The binders employed in this processing step were selected according to their homogeneity and their thermal and mechanical properties. Therefore, only the polyurethanes with molar ratio -NCO/-OH of 2:1 plasticized with 30 wt.% TEGDN were eligible to be used as binders for the rocket propellant formulations. Table 3 provides information regarding the composition of the new eco-friendly composite propellants (ECP) obtained in this study. All the formulations contained 57 wt.% PSAN with 200 μm granulation and 16 wt.% of PSAN with 50 μm granulation, as well as 1 wt.% of burn rate regulator Fe_2_O_3_.

#### 2.2.4. Characterization

The hydroxyl number of the polyols (I_-OH_) was determined according to ASTM-D4274-21 [39]. The acid number (I_A_) was determined according to ASTM D4662-20 [40]. The number-average molecular weight (M_n_) was quantified according to the “end -groups” method, using the acid number and hydroxyl number, without removing the free glycols, by using the following formula: Mn = (2∙56.1 × 1000)/(I_A_ + I_OH_) [41,42]. The FT-IR spectra of the synthesized polyols were recorded using a Spectrum Two FT-IR Spectrometer (PerkinElmer, MA, USA). The parameters used for the FT-IR ATR determinations were number of scans: 32; resolution: 4 cm^−1^; spectral range: 600–4000 cm^−1^. ^1^H-NMR spectra of the polyols were acquired with a Gemini Varian 300 MHz 300 MHz spectrometer (Varian Inc., Palo Alto, CA, USA). To conduct the ^1^H-NMR analyses, the samples were dissolved in 0.5 mL of deuterated chloroform (CDCl_3_). The viscosity of the samples was measured at 25 °C on a Rheotest 2.1 (Germany) device with coaxial cylinders. A shear rate varying from 1 to 1400 s^−1^ was applied for all samples. The thermal properties of the synthesized polyurethanes were investigated using DTA and TGA analyses. DTA OZM 551 Ex Differential Thermal Analysis System (OZM Research, Hrochův Týnec, Czech Republic), with Meavy dedicated software, was used for the evaluation of the influence of each component on the thermal properties of these new materials. Each test was performed on 35 mg of sample heated between 25 °C and 300 °C with a 5 °C/min heating rate, according to STANAG 4515 [43]. The thermogravimetric analysis (TGA) of the synthesized polyurethanes was executed using a Netzsch TG 209 F3 Tarsus instrument (NETZSCH, Selb, Germany). The experiments were performed at a heating rate of 10 °C/min under nitrogen flow, where samples of approximately 4 mg were heated from ambient temperature 25 °C to 700 °C. The morphology of the polyurethane films and the distribution of the main elements have been investigated by means of SEM-EDX (scanning electron microscopy) using a Tescan Vega II LMU SEM instrument (TESCAN, Brno, Czechia) at 10 keV acceleration voltage, under high vacuum. To ensure good data acquisition, the samples were sputter-coated with gold. The distribution of the metallic particles inside the polyurethane matrix of the rocket propellant composite was studied through micro-CT (μCT) method. Thus, the SkyScan micro-CT was attached to the previously mentioned Tescan Vega II LMU SEM instrument. Samples were examined as follows: exposure time, 4 s per projection at electron beam currents of 100 nA; accelerating voltage, 30 KeV; step size, 1°; scanning time, 24 min. The reconstructed images were obtained using the NRecon program by using float-point data values for internal calculations during reconstruction, which allow the operator to define the density window as a range of reconstructed values. The reconstruction results were visualized with the aid of the program DataViewer^®^ 2D/3D Micro-CT Slice Visualization (Micro Photonics Inc., Allentown, PA, USA). Tensile tests were carried out by using a 710 Titan 2 Universal Strength Tester by James H. Heal and Co. Ltd. testing machine (Richmond Works Halifax, UK). The tests were carried out according to the international standard ISO 37: 2011(E) [44]. Polyurethane samples were prepared by cutting tensile specimens (standard dumbbell: 5 mm width of the narrow parallel part, 100 mm total length, the distance between gauge marks 20 mm). Tests were performed at a rate of extension of 500 mm/min, starting from 50 mm jaw separation (plain jaw faces). For each polyurethane film, five tensile tests were performed, and the average value was reported. The low strain rate compression tests of the composite propellant were performed on an Instron 2519-107 Universal Test Machine (Instron, Norwood, MA, 02062-2643, USA). Cylindrical specimens, with a diameter of 20 mm and a length of 20 mm, were tested at a compression rate of 50 mm/min. Each test was repeated three times. Dynamic mechanical analysis (DMA) tests were carried out on a Discovery DMA 850 apparatus from TA Instruments (New Castle, DE, USA). Samples were analyzed in single cantilever mode, frequency 1 Hz, on a temperature ramp between −100 °C and 50 °C, using a heating rate of 5 °C/min maintained by cooling with liquid nitrogen. On the same instrument, 3-point bending clamp was installed to evaluate the sample sheets during a bending process consisting of successive stress, amplitude varying from 1 to 100 µm, 1 Hz, initial preload force 0.01 N, at 25 °C. The measurements of the frequency-dependent shear modulus were performed on the DMA 850 instrument with shear-sandwich clamps, at frequencies varying from 0.01 Hz to 10 Hz, 20 µm amplitude at 25 °C. For the single cantilever temperature ramp and 3 point bending analyses performed on DMA 850, the specimens were cut from the propellant cylinders in rectangular shapes with the following dimensions: 60 mm × 12.5 mm × 3 mm; for the shear-sandwich set-up, they were cut in square shapes of 10 mm × 10 mm × 1.5 mm. The experimental tests regarding the burning mechanics of the new environmentally friendly rocket propellants were conducted on a subscale rocket motor TRM35 (OZM Research, Hrochův Týnec, Czech Republic). The specimens were designed as cylinders with inner hole with the following dimensions: 40 mm × 12 mm × 45 mm (outer diameter × inner diameter × length).

## 3. Results and Discussion

**Scheme 3:**
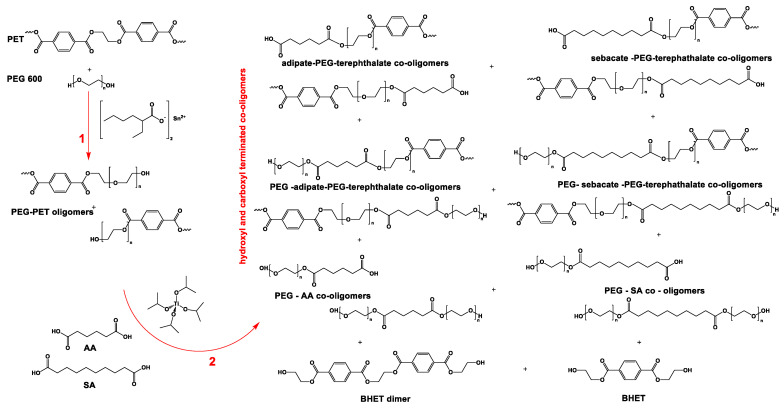
Hypothetical scheme comprising the main probable degradation products obtained through the two-step catalytic degradation of PET.

The degradation of PET through glycolysis involved the use of PEG600, employed in the first step, followed by the addition of AA and SA in the second step. The AA and SA were introduced in the second step of the reaction to ensure the consumption of the unreacted PEG600, while also serving as chain extenders and maintaining the low viscosity of the final reaction products mixture. Another purpose was to ensure a high concentration of OH-ending groups for the synthesis of the polyurethane binders. Scheme 3 illustrates only the main, hypothetical reaction products that resulted from PET, because it is difficult to depict all the combinations that may arrive from this situation.

Before proceeding with the synthesis of the polyurethane binders, a thorough analysis of the synthesized polyols was imperative. Thus, the products obtained through the two-step catalytic degradation of PET, together with the blends obtained with the commercial polyol and with the energetic plasticizer, were investigated through various analytical techniques; ^1^H-NMR and FT-IR were employed for the evaluation of the chemical structure of the polyester-polyol, while the titration methods offered information about the hydroxyl number and acid number. Rheological properties were also investigated, because the viscosity of the polyol blends directly influences the polyurethane synthesis (ease of mixing the components, reaction rate, homogeneity of the obtained materials) and the preparation of the final composite formulation. 

**Figure 1:**
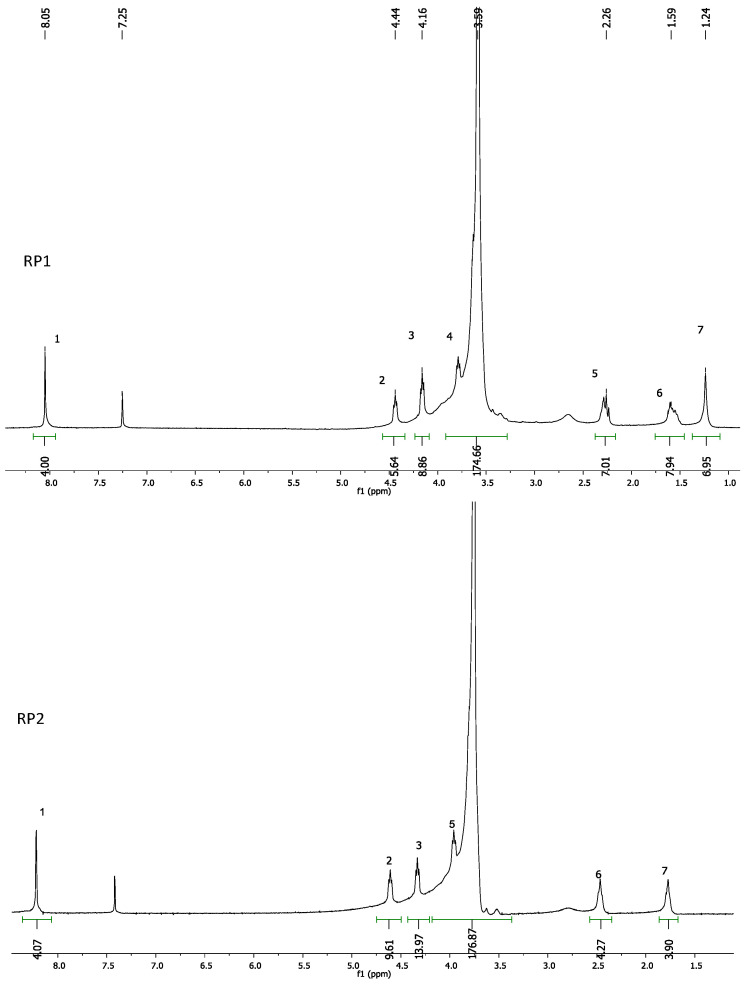
^1^H-NMR spectra of the polyol mixtures that resulted from the degradation of PET.

Figure 1 illustrates the ^1^H-NMR spectra of the polyols that resulted from the degradation of PET (RP1 and RP2). In both cases, the peaks labeled with 1 can be assigned to the protons from the aromatic rings (δ_H_ = 8.05 ppm, singlet) from the terephthalates moieties derived from PET, and the peaks labeled with 2 and 3, respectively, and 5 and 6 can be attributed to the protons from methylene groups adjacent to -COO groups (δ_H_ = 4.44 ppm, triplet and δ_H_ = 4.16 ppm, triplet, respectively, and δ_H_ = 2.26 ppm, multiplet and δ_H_ = 1.59 ppm, multiplet), while peak 4 (δ_H_ = 3.8 ppm, triplet) can belong to the protons adjacent to -OH groups, and peak 7 (δ_H_ = 1.24 ppm) can be attributed to the protons from methylene groups adjacent to other methylene groups. The intense peak from 3.59 ppm can be attributed to PEG fragments. Figures S1–S4 from the “Supplementary Material” file illustrate the ^1^H-NMR spectra of the polyol blends that resulted from mixing RP1 and RP2, respectively, with SET and with SET+TEGDN. In comparison with the plots obtained for the neat polyester-polyols derived from PET (RP1 and RP2), the plots from the “Supplementary Material” file contain two additional peaks in the 5.4–5.5 ppm region, specific for the tertiary proton of the -CH2CHCH2- backbone of SET (this commercial polyol is mainly based on castor oil) and one new peak at δ_H_ = 4.61 ppm, specific for the protons adjacent to nitro groups in TEGDN.

**Figure 2:**
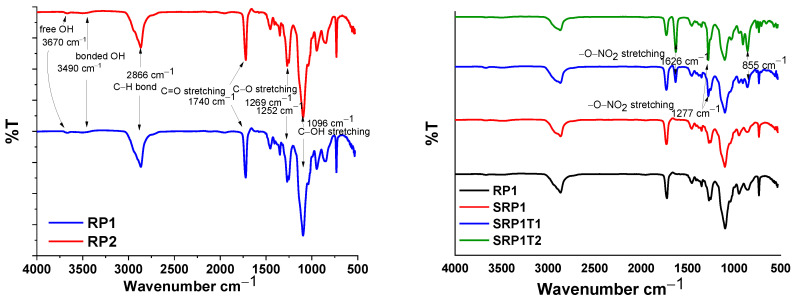
FT-IR spectra of RP1 and RP2 polyols and mixtures SRP1, SRP1T1, and SRP1T2.

In all the polyol mixtures (polyester-polyols obtained from the degradation of PET (RP1 and RP2) and polyol mixtures (SRP1 and SRP2, SRP1T1 and SRP1T2) (see details on composition in Table 1), FT-IR spectra confirmed the presence of free hydroxyl groups by the presence of a peak at the 3667–3670 cm^−1^ region. The presence of intermolecular bonded hydroxyls was also visible through a broad peak situated in the 3475–3490 cm^−1^ region. The intense peak that can be observed around 1740 cm^−1^ can be attributed to C=O stretching from ester groups. C-OH stretching vibrations are visible in the 1097–1100 cm^−1^ region. The peaks from 1269 cm^−1^ and 1252 cm^−1^ can be assigned to C-O bonds, while 2866 cm^−1^ and 2945 cm^−1^ can be assigned to C-H bonds. C-H bending can be evidenced through the peaks present at 1454 cm^−1^ and 1350 cm^−1^. For the samples containing TEGDN (SRP1T1 and SRP1T2), three additional peaks can be observed at 1626 cm^−1^, 1277 cm^−1^, and 855 cm^−1^, which represent the -O-NO_2_ stretching vibrations. The data can be observed in Figure 2. The RP2 mixture spectra are presented in Figure S5 in the “Supplementary Material” file. 

The number-average molecular weight (M_n_) of the compounds that resulted from the degradation of PET was estimated based on the experimental values obtained from the hydroxyl number and the acid number. The results are presented in Table 4. As can be observed, when the ratio of glycol/PET (RP1) was higher, the numerical-average molecular weight (M_n_) obtained for the glycolyzed products was lower. Since these products will be included in the synthesis of the polyurethane binders designed for energetic mixtures, the results presented in Table 4 are useful for further calculations (e.g., calculation of the amount of MDI necessary for the synthesis of polyurethane binders).

**Figure 3:**
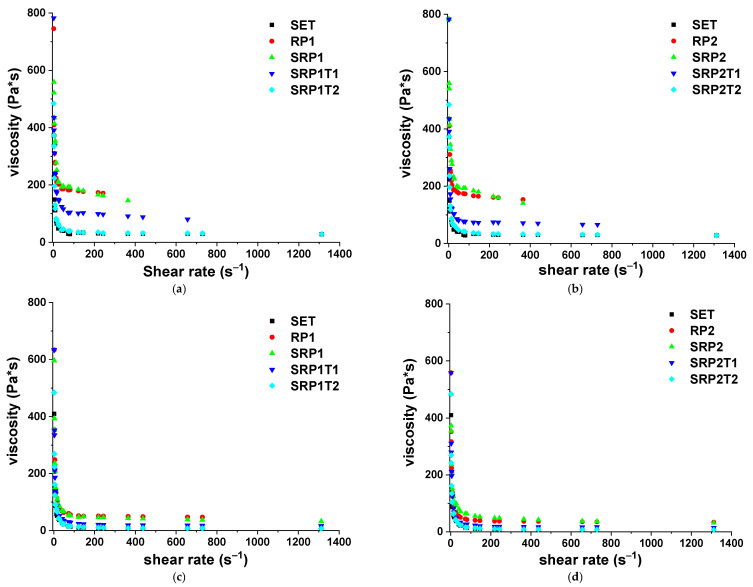
The effects of SET and TEGDN upon the viscosity of polyester-polyols.

An important aspect for this type of application (polyurethane-based binders for rocket propellants) needs to be underlined: when the PET degradation reaction is completed, the free glycols should not be separated. Thus, they offer the possibility to maintain a low viscosity for the glycolyzed products mixture, which is an important factor for the synthesis of polyurethane binders. The presence of these unreacted glycols led to lower viscosities of the RP1 and RP2, also demonstrated through rheological measurements illustrated in Figure 3a–d. The neat commercial polyol (SET) exhibited lower dynamic viscosities than the polyol mixtures that resulted from the degradation of PET (RP1 and RP2). The addition of the energetic plasticizer to the polyester-polyol blends can significantly improve the performance of these rocket propellants. Therefore, analyzing its influence on the physical and chemical properties of these energetic composites is of great importance.

The plots illustrated in Figure 3a–d describe the behavior of the polyol mixtures at 25 °C (Figure 3a,b) and 50 °C (Figure 3c,d), with the increase of the shear rate. As can be observed, RP1 and RP2 do not seem to exceed some of the tolerable limits [1,2] for this type of application, their viscosities allowing them to be easily mixed with the solid components of the rocket propellants. Moreover, the addition of SET and TEGDN led to even lower viscosities, facilitating the synthesis of homogenous binders. The viscosity of the mixtures SRP1 and SRP2, which resulted from the addition of the commercial polyol (SET) to the recycled polyols (RP1 and RP2), was slightly increased in comparison to neat RP1 and RP2. However, this aspect does not represent a problem for this type of application, because the addition of TEGDN results in a decrease of the polyol mixture’s viscosity. It was observed that the viscosity of the polyol blends diminished with the extent of TEGDN added: the dynamic viscosity of SRP1T1 was higher than for SRP1T2. The polyol mixtures based on RP2 exhibited the same behavior: the dynamic viscosity of SRP2T1>SRP2T2. A lower viscosity of the polyol mixture will facilitate the mixing process of the rocket propellant composite, at least in terms of rheological properties.

To obtain specimens with a certain geometric shape, specific for each investigation method employed for this study, after following the three steps of polyurethane synthesis illustrated in Scheme 1, the freshly prepared liquid polyurethanes were poured into glass vessels and placed in a vacuum oven. During the curing process, the time was recorded, and the process of surface hardening was examined periodically. According to the observations summarized in Figure S6 from the “Supplementary Material” file, we can conclude that the curing time of polyurethane samples is inversely proportional to the -NCO/-OH ratios. More precisely, when the concentration of MDI increased, the curing time decreased. All three types of SET-MDI films successfully incorporated a large amount of TEGDN, while in the case of the polyurethanes based on the polyols that resulted from PET, some problems were identified for PU_11T1, PU_11T2, PU_12T1, and PU_12T2, where the reaction seemed incomplete. In the case of PU_22T2 and PU_32T2, the surfaces had an irregular aspect. Moreover, the color changed and the ‘air-bubble‘ trapping that was observed in the case of PU_2, PU_3, PU_21, PU_31, PU_22, and PU_32, was diminished as we increased the amount of TEGDN added to the polyol blends. A brief description of the cured samples and curing time (T_C_) for each formulation is depicted in Figure S6 in the “Supplementary Material” file.

The visual inspection of the cured polyurethane films (illustrated in Figure S6 in the “Supplementary Material” file) offered evidence about the potential polyol blends that can serve as candidates for the polyurethane binders. Based on their visual aspect, for a detailed investigation, polyurethane films having 3:2 and 2:1 -NCO/-OH ratios, with and without TEGDN, were chosen to be subjected to SEM-EDX analysis. These examinations were performed only for RP-based polyurethanes, because the aim of this study consisted in developing eco-friendly binders for “green” rocket propellants. The morphological results provided by SEM analysis can be found in Figure S7 in the “Supplementary Material” file. The visual inspection of the polyurethane films revealed that the surfaces of the samples based on RP2 (samples PU_22T2 and PU_32T2) were irregular. Therefore, an elemental mapping of carbon, nitrogen, and oxygen, with the aid of an energy dispersive X-rays (EDX) technique, was necessary for a better understanding of the interaction between the elements. According to EDX images, displayed in Figure S8, all the polyurethane formulations based on RP1 showed good homogeneity. The same can be observed for PU_22 and PU_32 samples. However, in the case of PU_22T2 and PU_32T2, there is an obvious lack of homogeneity, probably induced by the incompatibility of TEGDN with the polyol blends containing RP2. Therefore, for the next steps of our study, based on the information obtained through SEM-EDX analysis, we decided to employ only the polyol formulations containing RP1.

**Figure 4:**
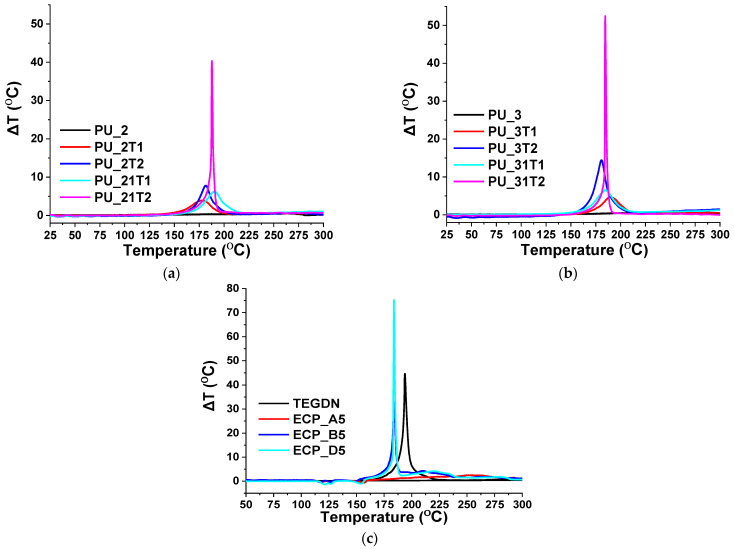
DTA thermograms for polyurethane films: (**a**) 3:2 -NCO/-OH group molar ratio; (**b**) 2:1 -NCO/-OH group molar ratio; (**c**) neat TEGDN and composite rocket propellants.

The thermal behavior of polyurethane films (with and without the energetic plasticizer, TEGDN) was investigated by TGA and DTA analytic techniques. Due to safety reasons, the rocket propellant composite formulations were analyzed only by DTA means. Differential thermal analysis (DTA) gives complementary information about the thermal properties of the synthesized materials. Typical DTA thermograms for the polyurethane films, based on 3:2 and 2:1 -NCO/-OH group molar ratios, are shown in Figure 4a,b. Figure 4c displays a comparative plot between neat TEGDN and the rocket propellant composites obtained in this study. There is only one exothermic peak of great significance for both films and composites. The decomposition temperature attributed to this peak varies in the interval of 190–200 °C; detailed values, for each formulation, are summarized in Table S3 from the “Supplementary Material file” file. Based on the DTA analysis, this high exothermic peak, present in all thermograms of the formulations containing TEGDN (Figure 4c), corresponds to the decomposition temperature of this energetic plasticizer [36]. After evaluating the thermograms illustrated in Figure 4 together with the values summarized in Table S3 from the “Supplementary Material file” file, it can be observed that the higher compatibility between RP1 and TEGDN (confirmed by SEM-EDX) led to a more efficient energetic binder, which can be demonstrated by the existence of a higher and sharper peak of decomposition for the rocket propellant ECP_D5 (comprising a polyurethane binder based on RP1, molar ratio 2:1, sample PU_31T2). The positive influence of RP1 on the thermal performances of the synthesized materials can be observed in all the thermograms containing RP1 and TEGDN (Figure 4a–c), samples PU_31T2 and ECP_D5. Therefore, we can conclude that the optimal polyurethane binder, in terms of thermal performances of the rocket propellant, is represented by the polyurethane based on RP1 (sample PU_31T2), which seems to enhance the exothermic efficiency of the energetic formulation.

**Figure 5:**
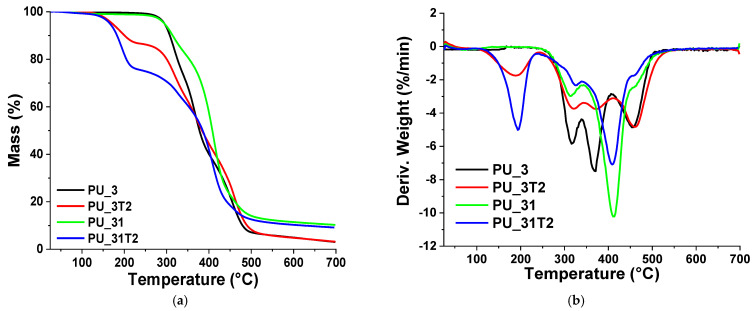
(**a**) TGA and (**b**) DTG profiles of the polyurethanes.

Moreover, the sharpness of the characteristic exothermic peak gives indications about reaction kinetics, being related to the rate of reaction. It is commonly known that the energetic materials undergo fast exothermic degradation transformations, which also occur with our rocket propellant composites, a necessary characteristic for obtaining efficient propulsion. This positive effect was not present when PU_3T2 was employed in the synthesis of the rocket propellants (sample ECP_B5). As can be observed in Figure 4, the peak height of the composite ECP_D5 (based on polyurethane containing TEGDN, sample PU_31T2) is higher than the peak height of neat TEGDN. Therefore, this gives clear evidence of the positive effect that RP1 has on the energetic formulation, enhancing the exothermic decomposition process, which can be considered beneficial and desirable for this type of application. The samples containing only the commercial polyol (SET), presented here for comparison, namely the samples named here PU_3 and ECP_A5, displayed lower values in terms of thermal performances. Therefore, we can conclude that the most efficient polyurethane formulation that can be employed as an energetic binder for the rocket propellants involved both commercial (SET) and recycled (RP1) polyols together with the energetic plasticizer (TEGDN, 30 wt.%). Namely, the optimal polyurethane binder was the sample coded with PU_31T2. Since in the DTA analysis the polyurethane films based on a 2:1 -NCO/-OH molar ratio (sample PU_31T2) displayed the best values for these rocket propellants, thermogravimetric analysis was performed on the samples based on SET (samples PU_3 and PU_3T2) and SET+RP1 (samples PU_31 and PU_31T2), to obtain complementary information regarding the thermal properties of the polyurethane films. Figure 5 displays TGA (a) and DTG (b) curves for the new synthesized polyurethane films. As can be observed in Figure 5 and Table S4 from the “Supplementary Material” file, the decomposition process of the polyurethane films based on SET (sample PU_3) began slightly earlier than in the case of the polyurethane films obtained with SET+RP1 (sample PU_31). This trend was not similar for the samples containing TEGDN, because the decomposition process for sample PU_31T2 began earlier due to better compatibility and miscibility of RP1 with the energetic plasticizer (as already confirmed by SEM and DTA analyses). The peaks displayed in the DTG curve offer information about the succession of the degradation phases of the polyurethane films. The first peak, situated around 190 °C, can be attributed to the decomposition of TEGDN embedded in the polyurethane matrix, while the peaks that appeared starting at 300 °C can be assigned to the degradation of the polyurethane matrices.

**Figure 6:**
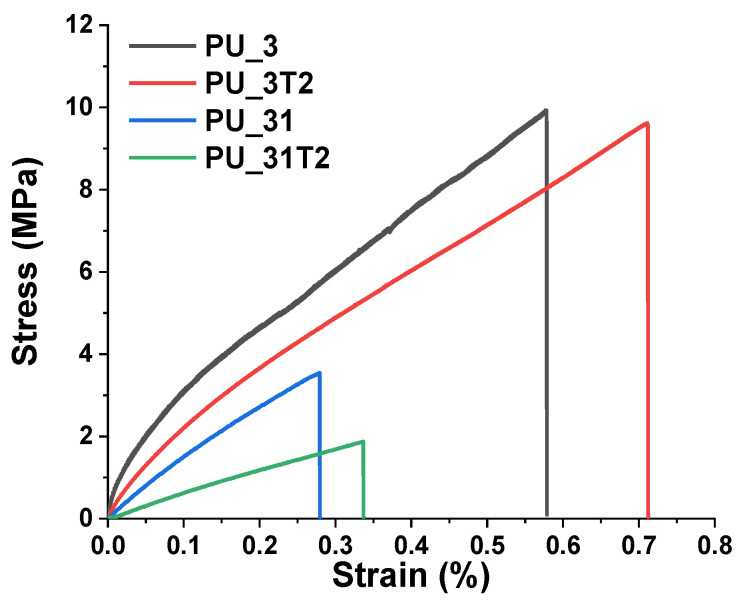
Tensile stress–strain plots of polyurethane films.

**Figure 7:**
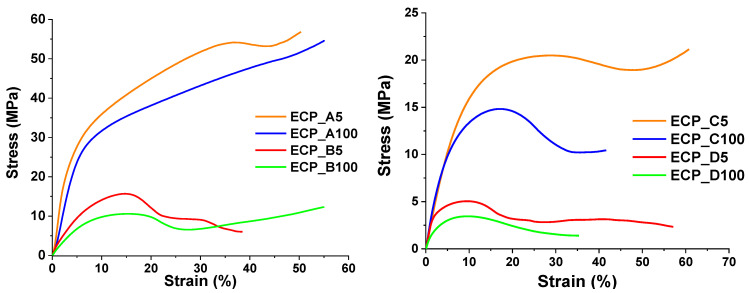
Compressive stress–strain profiles for composite rocket propellants.

Table S5 from the “Supplementary Material” file summarizes the weight loss percentage, at certain temperatures, for the polyurethane binders. As can be observed, those containing TEGDN, samples PU_3T2 and PU_31T2, displayed earlier a significant weight loss than the polyurethane matrices without the energetic plasticizer (samples PU_3 and PU_31). The mechanical properties of the polyurethane matrix directly influence the mechanical behavior of the propellant grains. Thus, the commercial and recycled polyol-based polyurethanes (with and without TEGDN) were subjected to tensile stress. The comparative tensile stress–strain profiles of PU_3, PU_3T2, PU_31, and PU_31T2 are depicted in Figure 6, while in Figure S9 from the “Supplementary Material” file can be found the stress–strain curves of each specimen of the polyurethane formulation. As can be observed, the maximum values of tensile stress and strain were reached by the polyurethanes based on the commercial polyol, samples PU_3 and PU_3T2. Even though, theoretically, samples PU_31 and PU_3 should display similar mechanical behavior (both are based on Setathane (SET)), tensile tests revealed lower mechanical resistance for PU_31 polyurethanes. The difference between the mechanical properties of these two analogue matrices (samples PU_3 and PU_31) appeared due to the introduction of RP1 during synthesis. This polyol blend (SRP1) was obtained by mixing SET (commercial polyol) with RP1 (polyol that resulted from the degradation of PET). Besides the long aliphatic chains of SET (which is a branched, castor oil-based polyol [45]), it contains multiple shorter chains belonging to RP1, which led to a decrease of the overall mechanical resistance of these polymeric films. The introduction of an energetic plasticizer in the polyurethane network, which is compatible with the other reactants employed for the synthesis (RP1, SET, and MDI), is imperative for enhancing the performance characteristics of the correspondent rocket propellant. As can be seen from Figure 6, the addition of TEGDN led to a higher elongation of the tested specimens and thus an increase of strain values, but the stress values were slightly decreased. The Young’s modulus followed the same trend, decreasing with the addition of RP1 and TEGDN. Explicit values for the Young’s modulus, maximum tensile stress, and strain of the synthesized polyurethane films are presented in Table 5. The mechanical properties of the rocket propellant composites are not influenced only by the nature and concentration of the polyurethane binder, but it also depends on the properties and concentration of the solid components (oxidizer, metallic fuel). Thus, to investigate the influence of the metallic fuel granule size on the mechanical properties of the newly synthesized composite rocket propellants, different propellant formulations were subjected to tensile tests (Table 6) and compression (Figure 7 and Table 7). 

Compression deformation at the breaking point of the composite propellants, as a function of the metallic fuel (aluminum) particle size, is illustrated in Figure 7. During the tests, the percentage of binder used was the same for all samples. Hardness appears to be directly influenced by particle size and binder type used. The most rigid sample is ECP_A5, due to PU_3 and small particles (<5 μm), while the most elastic is ECP_D100, based on PU31_T2 and large particles (100 μm), as shown in Table 7 for a strain value around 0.2%. Comparing formulations with similar grain sizes, the stress–strain differences appear due to the use of RP1 and TEGDN. Thus, before adding TEGDN, ECP_A5 and ECP_A100 showed a compressive stress resistance four times higher than that of ECP_B5 and ECP_B100, respectively. Similar behavior was distinguished for ECP_C5, ECP_C100, ECP_D5, and ECP_D100, based on RP1. The use of RP1 decreases the resistance only twice compared to those that did not contain it. As anticipated, the addition of the energetic plasticizer, TEGDN, has a significant impact on the compression behavior of these materials.

Since the formulations based on small particle granulation exhibited higher compression resistance, the tensile tests were performed only for this category (aluminum particles size < 5 μm). The composite tensile test results are shown in Figure S10, except for ECP_A5, because its mechanical resistance exhibited the limits of traction force of the instrument employed for this analysis. As can be observed in Table 7, the stiffness of the rocket propellant specimens varied with the modification of the grain sizes and their composition. Specifically, the rocket propellants based on RP1 displayed poorer mechanical resistance than those based only on SET. On the other hand, the composite propellants containing smaller grain sizes (samples ECP_B5, ECP_C5, and ECP_D5) of the solid components displayed better mechanical resistance, due to a better arrangement of these grains inside the polymeric matrix. The results indicate that the mechanical characteristics of the new materials are closely related to their internal structure, homogeneity of the solids dispersion in the binder matrix, particles sizes, and good interaction of the organic binder with the grains of the inorganic oxidizer and metallic fuel. 

The mechanical behavior of these binders, such as elasticity, viscosity, hardness, brittleness, or rigidity, under a variety of stresses (for example pressure or traction) influences the mechanical properties of the rocket propellant composites. Even if the polyurethane matrices based on the commercial polyol (PU_3 and PU_3T2) displayed higher mechanical resistance than those containing the polyols from PET degradation (PU_31 and PU_31T2), we can conclude that these recycled materials possess a mechanical resistance suitable to be employed as binders in rocket propellants. Moreover, the selection of the polymeric matrix of the binder must take into consideration the advantages brought by the recycled polyols through their outstanding thermal properties. Thus, a compromise can be made, and despite their poorer mechanical resistance (which is situated within some tolerable values), the binders based on recycled PET can enhance the thermal performances of the rocket propellant and contribute to a less polluted environment. Moreover, its higher flexibility will ensure resilience, a better shock resistance, which is very important for maintaining the geometry of the propellant, preventing crack formation and adhesion failure.

**Figure 8:**
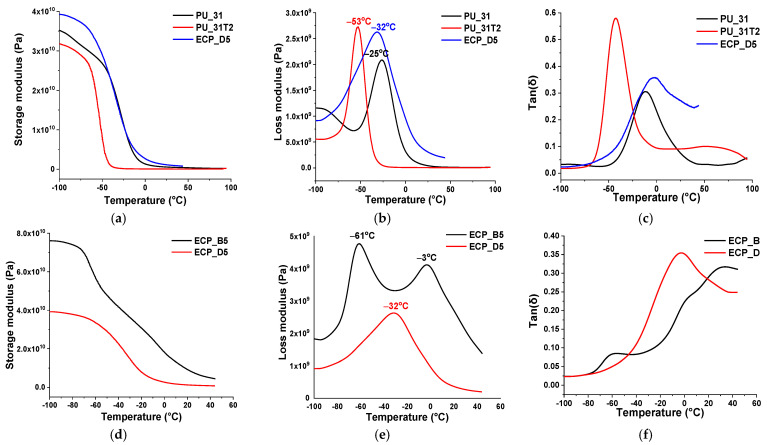
Dynamic mechanical analysis for polyurethanes and composite propellants. (**a**,**d**) Storage modulus for polyurethane films and for the corresponding composites; (**b**,**e**) Loss modulus for polyurethane films and for the corresponding composites; (**c**,**f**) Tan delta plots for polyurethane films and for the corresponding composites.

DMA analysis was aimed at investigating the thermo-mechanical properties of the synthesized polyurethanes and polymeric composites in a single cantilever bending mode (on temperature ramp from −100 °C to +50 °C or +100 °C), three-point bending mode (ambient temperature, +25 °C), and shear-sandwich configuration (ambient temperature, +25 °C). The rocket propellant composites were tested only up to +50 °C due to safety reasons [46], as they are energetic materials. Figure 8 illustrates a comparative plot of the storage modulus, loss modulus, and tan delta obtained in a single cantilever bending mode for the polyurethanes (Figure 8) based on polyols obtained from the degradation of PET (with and without TEGDN, namely PU_31 and PU_31T2). The DMA results for the corresponding polymeric composites are presented in Figure 8d–f: based on commercial polyol, ECP_B5; based on polyols recycled from PET, ECP_D5. According to STANAG 4540 [47], the maximum value of the loss modulus E″ peak of the composites for rocket propellants can be considered their glass transition temperature. Therefore, according to this standard for energetic materials, we can assume that the glass transition temperatures of our samples correspond to the maximum of the loss modulus E″ peak. Thus, PU_31 (the polyurethane based on polyols recycled from PET) displayed a glass transition temperature at around −25 °C, while its homologous polyurethane containing TEGDN (PU_31T2) displayed a lower T_g_ (−53 °C), due to its plasticizing effect. The rocket propellant composite (comprising the polyurethane based on polyols recycled from PET), ECP_D5, displayed a glass transition temperature at approximately −32 °C. In comparison with the neat polyurethanes, this transition is also influenced by the interactions that occur between the solid components of the rocket propellant composite and the polymeric matrix of the binder. The structure and the properties of the rocket propellant composites are influenced by the composition and ratio of the blended polyols employed for the synthesis of the binder. Thus, it can be observed that the composite containing only SET, ECP_B5, displayed two glass transition temperatures, due to a stronger segregation [48] of the hard and the soft segments of the polyurethane employed in this type of composite. In this case, the polymeric chains of SET (castor-oil-based polyol), possessing considerably higher lengths, will generate larger zones where soft segments will merge. Thus, the structuring of these polyurethanes will have a particular microscopic aspect, consisting of congregated hard segments (consisting mostly of aromatic regions originating from MDI, Desmodur^®^44V20L), which will look similar to “isles areas” in the soft polymeric matrix (consisting of long aliphatic chains originating from SET). This behavior can also be observed in SEM-EDX images illustrated in Figure S7. On the other hand, the recycled polyol blends did not lead to the same behavior as the polyurethanes, probably because in this case, soft segments are more homogenous and intercalated with hard segments due to the shorter length of the diols employed for the synthesis of these binders. Thus, the rocket propellant composites that contain binders based on polyurethanes obtained from recycled polyol blends displayed only one T_g_ point (−32 °C).

**Figure 9:**
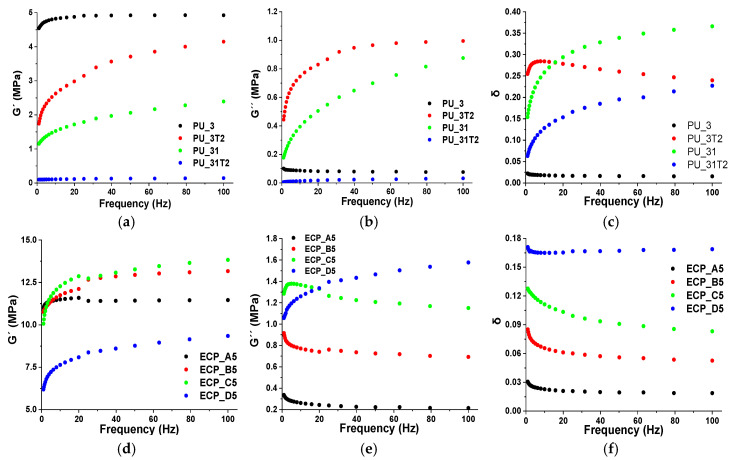
Shear storage modulus (**a**,**d**), shear loss modulus (**b**,**e**), and tan delta (**c**,**f**) profiles vs. frequency for synthesized polyurethanes and for the corresponding composite propellants.

Many properties of viscoelastic materials are dependent on time (frequency). The viscoelastic nature of polyurethane binders employed in this study requires consideration of their creep behavior during the design process of the rocket propellant composites. This variation is illustrated in Figure 9a–f, for the shear storage modulus, shear loss modulus, and δ, based on data collected in the shear-sandwich DMA set-up. As can be observed in Figure 9, the storage modulus presented a significant increase in the sample containing the commercial polyol and TEGDN (PU_3T2), indicating that this plasticized polyurethane has a great potential for storing the energy during a loading cycle [49].

Consequently, this parameter can be related to the shape recovery of this polymer during loading. Similar behavior was observed for PU_31, the polyurethane based mainly on polyols derived from PET. Therefore, we can sustain that these materials also possess a remarkable capacity for storing energy. This ability is important for the binders employed in the rocket propellant composites because, when the energetic charge/loading encounters a shear stress situation, the polyurethane binder absorbs the energy, thus preventing the accidental initiation of the energetic composite. The loss modulus, which characterizes the ability of the polyurethane binder to dissipate this energy through the internal molecular motions of the polymeric chains, displayed higher values for the PU_3T2 and PU_31 samples. Therefore, tracking the changes of the shear modulus as a function of frequency offers evidence about the “damping” behavior of these polyurethanes by describing the dissipation of mechanical energy through internal motion (loss modulus, tan delta). Comparing these mechanical properties of the synthesized polyurethanes with those obtained for the corresponding rocket propellant composites (Figure 9d–f), we can affirm that the composite based off the recycled polyol blends and TEGDN (ECP_D5) displayed the most significant increase in terms of storage and loss modulus. Thus, we can presume that this rocket propellant composite displayed better ability to store and disperse mechanical energy. Tan delta (δ) represents the ratio of the loss to the storage modulus, and it is often called damping, being a measure of the capacity of the dissipation of energy in a material under cyclic loadings. All the composites designed for use as rocket propellant exhibited similar frequency-dependent damping behaviors. These polyurethane-based composites displayed slightly decreased values for tan delta at higher frequencies. This behavior can be explained by the gradual slippage exhibited by the main backbone chain of the polyurethane binders while shear stress was applied. Their damping capacity reflects their ability to dissipate mechanical energy. Therefore, we can conclude that they will be able to reduce the vibration amplitude significantly. The damping properties of the solid components from these rocket propellants conjoined with the remarkable damping properties of the employed polyurethane matrices (Figure 9c), which led to high-performance energetic composites, are suitable to be safely used as rocket propellants.

**Figure 10:**
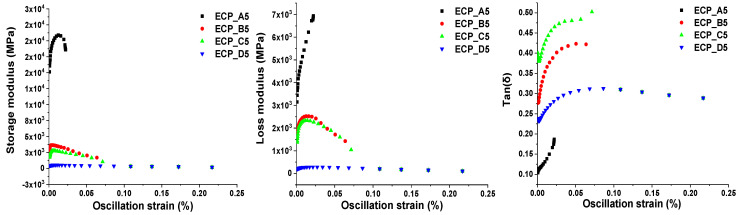
Bending behavior of the composite rocket propellants.

The rocket propellant composites (based on the synthesized polyols) were also analyzed using a three-point bending clamp. This is a typical flexural test that uses three identically sized cylindrical rollers to bend the sample. The three-point bending mode was employed as a method of analysis of the rocket propellant composites because, according to literature data, this type of deformation is usually considered “cleaner” than either the single/dual cantilever or tension modes, since clamping effects are eliminated [50]. For each sample, the oscillation amplitude was incrementally increased from 1 µm to 500 µm. As can be observed in Figure 10, the composites based on recycled polyols and TEGDN (ECP_D5) displayed higher flexibility, being the only rocket propellant composite that did not break during the bending test. The other energetic composites broke earlier because they were more rigid. An appropriate flexibility of these materials ensures the ease of charging the rocket motor with the composite propellants and guarantees that these energetic composites will maintain their integrity when the rocket motor is subjected to external mechanical stress.

**Figure 11:**
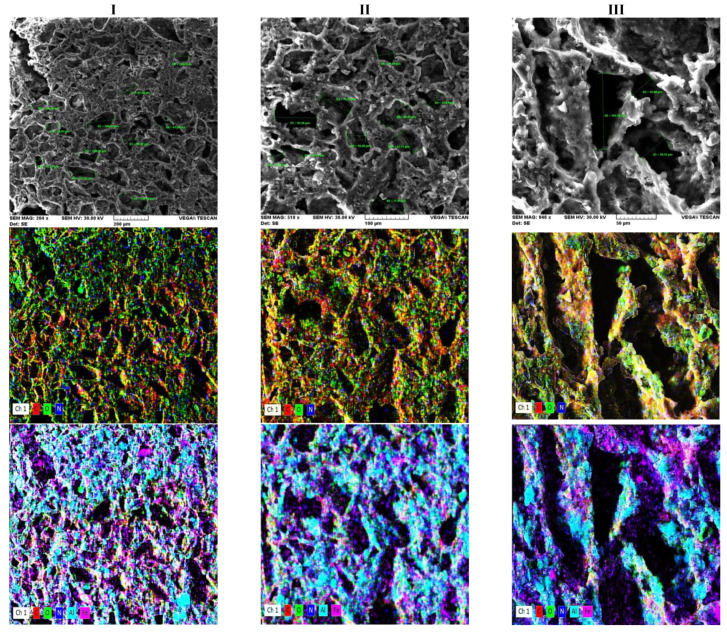
SEM and EDX analysis of the porous ECP_D5 formulation (after oxidizer extraction) at different scales: (**I**) 200 μm; (**II**) 100 μm; (**III**) 50 μm.

The highly aqueous solubility of PSAN employed in these rocket propellant composites allowed its extraction from the polymeric matrix and the assessment of the inner morphology of the energetic composites pressed in a cylindrical geometry. For this goal, small transversal slices of ECP were cut and placed in an aqueous solution until the oxidizer particles completely dissolved and migrated from the polyurethane matrix into the water solution. The samples were recovered after 6 h and dried at 50 °C for 12 h. The morphology of the resulted specimens was evaluated through the SEM-EDX technique. Figure 11 shows the SEM-EDX morphological images of the “oxidizer free” composite samples, investigated at different scales. According to these results, the voids that can be observed in these images correspond to the spaces that were previously occupied by the grains of the oxidizer. The dissolution of the oxidizer in water leads to a porous polyurethane matrix (still containing the metallic component). This experiment allows us to evaluate the way that the oxidizer solid grains are dispersed inside the polyurethane matrix, this being an important aspect for the investigation of the burning rate behavior inside the motor rocket chamber. Furthermore, based on the EDX analysis depicted in Figure 11, the distribution of aluminum and iron oxide particles inside the polyurethane matrix can be evaluated. Figure S12 from the “Supplementary Material” file presents the SEM-EDX morphological analysis of the surface for the composite formulations with oxidizer and both types of polyurethanes (based on commercial polyol, PU_3, and based on polyols obtained from PET degradation, PU_31, 2:1 molar ratio -NCO/-OH). Figure S13 and Table S1 illustrate the elemental composition of the “oxidizer free” composite samples. For comparison, Figures S14 and S15, and Table S2 respectively illustrate a morphological characterization of a pressed solid mixture of an oxidant and metallic fuel, without a binder. In the absence of the binder, the mechanical resistance of this pressed mixture is almost inexistent, being a very friable structure.

Based on this SEM-EDX evaluation of the rocket propellant composites, we can conclude that the solid grains of the oxidizer and metallic fuel are uniformly distributed inside the polyurethane binder matrix, which ensures a good cohesion between all the solid components, conferring proper explosive and mechanical characteristics.

**Figure 12:**
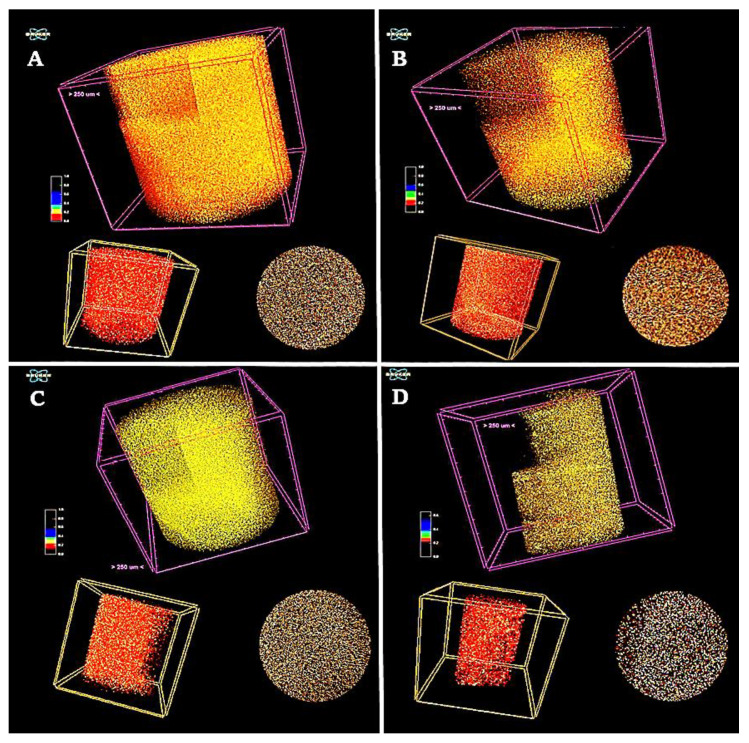
Micro-CT images (250 μm voxel size) of composite formulations: (**A**) ECP_A5; (**B**) ECP_B5; (**C**) ECP_C5; (**D**) ECP_D5.

As mentioned above, our composites, being specifically designed to be employed as rocket propellants, require a uniform dispersion of the solid components inside the polymeric matrix to ensure a constant burning rate. Thus, for obtaining complementary information to the SEM-EDX analyses presented above, the synthesized composites were also subjected to μCT analysis to evaluate the 3D distribution of the solid components inside the rocket propellant composites. As can be observed in Figure 12, all the composites displayed a homogenous three-dimensional distribution of the solid components in the polyurethane matrix. There are some slightly notable differences between the four rocket propellant composites illustrated in Figure 12, due to the addition of TEGDN and also due to the addition of the recycled polyol, which led to a less dense network of hard nanodomains inside the polyurethane matrix than the commercial polyol.

**Figure 13:**
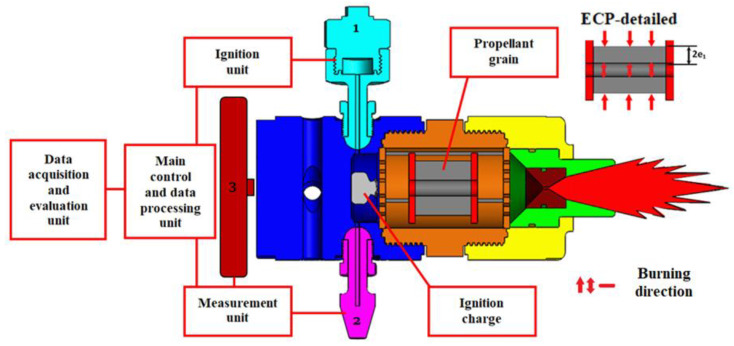
Subscale rocket motor set-up: (**1**) ignition; (**2**) pressure transducer; (**3**) thrust transducer (e_1_: burning propellant thickness).

The evaluation of the ballistic performances of these new rocket propellants based on PSAN, Al, and polyurethane matrices synthesized from polyester-polyols derived from PET degradation cannot be complete without confirming its applicability in a firing set-up. For this reason, a series of static experimental firings was performed with the ECP_D5 composite on a subscale rocket motor (SRM) TRM-35 to determine its performance characteristics. The experimental system was designed to investigate the burning behavior of the composite propellant for small-grain rockets before introducing them into a large-scale launching system. Figure 13 shows the experimental set-up used in the test. The propellant grain was wet pressed and cured in a cylindrical geometry with the inner bore, with obstructed front and rear ends, providing a neutral burning [2] from the inner and outer surface of the cylinder, as can be seen in detail in Figure 13. A structural view of the subscale rocket motor stand burner and the rocket propellant cylindrical composites is shown in Figures S16 and S17 from the “Supplementary Material” file. The ignition was ensured by a small amount of pyrotechnic composition, presented in Figure S18. To ensure the pressurization of the combustion chamber and the propellant grain ignition, the nozzle was equipped with an aluminum membrane that breaks after ignition. Figure S19 provides a view of the eco-friendly rocket propellant at the convergent section nozzle and the shape of the flame generated during an outdoor combustion session.

**Figure 14:**
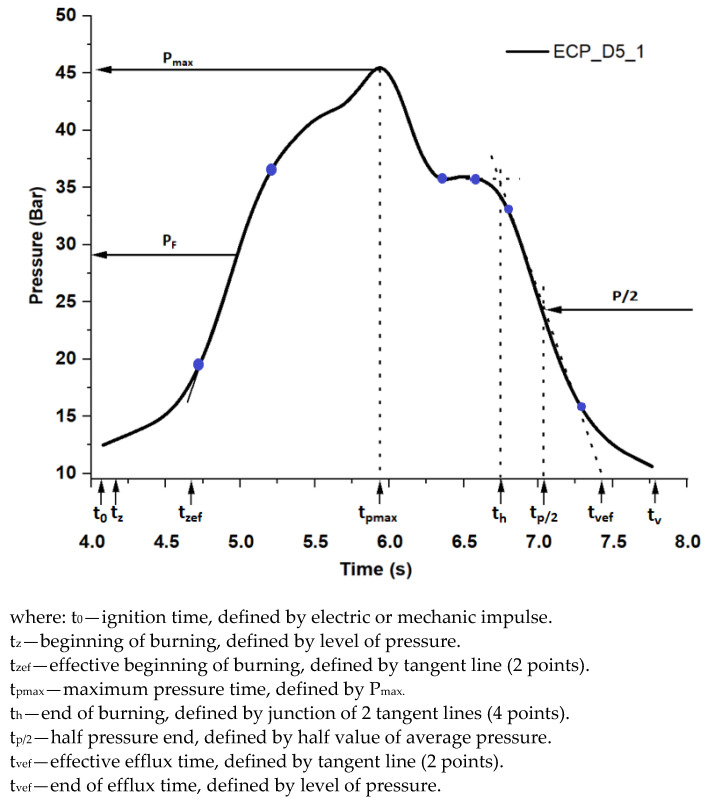
The burning rate profile and characteristics for the developed propellant.

The burning characteristics for the environmentally responsible rocket propellant obtained in this study are illustrated in Figure 14. Although the aspect of the burning profile pressure vs. time does not perfectly match the theoretical burning profiles of rocket propellant, due to the low scale of the experiment, we were able to emphasize that it provides promising results for this type of application. Thus, the average value of the pressure $\;\bar{(p_{th}})$, calculated with Equation (1), was 39.23 bars, which indicates good premises for this type of propellant, being in accordance with the values presented in the literature [50]. An average combustion rate ($\bar{u_{th}}$) of 2.78 mm/s was calculated using Equation (2), which is in the same range with the burning rate of ammonium perchlorate-based composite propellants [51]. The burning profile of the sample indicates a progressive burning at the beginning and then a regressive burning, probably due to the erosion of the propellant grain at the obstructed front/end surfaces and the transition towards a spherical geometry.

(1) $$\bar{p_{\mathrm{th}}}=\frac{1}{t_{h}-t_{z}}\overset{t_{h}}{\underset{t_{z}}{\int }}p\left(t\right)\mathrm{dt}$$

(2) $$\bar{u_{\mathrm{th}}}\left(\bar{p_{\mathrm{th}}}\right)=\frac{e_{1}}{t_{h}-t_{z}}$$

where: e_1_ is the burning thickness, as presented in Figure 13 and (t_h_ − t_z_) = t_b_ is the burning time interval.

## 4. Conclusions

Novel polyurethane binders and their applications in future environmentally responsible composite rocket propellants were investigated. To highlight the advantages brought by this new “green” approach, in comparison with state-of-the-art HTPB binders (extensively utilized nowadays in this field), we further summarized the most important achievements of this study. The polyurethane-based binders were synthesized using polyester-polyols obtained from catalytic degradation of recycled PET, commercial polyols, and the energetic plasticizer TEGDN. To demonstrate that the polyurethanes are suitable for this type of application, they were subjected to structural and rheological characterization (^1^H-NMR, FT-IR, and viscosity analysis), while the new composite propellants were subjected to morphological, thermal, and mechanical characterizations using various analytical techniques (SEM-EDX, DTA, TGA, DMA, µCT, and tensile and compression tests). Moreover, the composite propellant formulations developed were analyzed in terms of ballistic performances by real firing tests in a subscale rocket motor.

^1^H-NMR and FT-IR characterizations confirmed that the synthesized polyester-polyol has the appropriate chemical structure, while their viscosity follows the requirements imposed in the development of propellants. SEM-EDX and μCT analyses proved the homogenous dispersions of the solid load inside most of the composites. The uniformity of the oxidizers and fuel distribution inside the polymeric matrix was also indicated by the continuous combustion of the propellant grain, thus improving the energetic performances of these composites. TGA measurements indicated that the presence of TEGDN lowered the decomposition temperature of the polyurethanes, an aspect also confirmed by DTA investigations. However, the polyurethanes possess good thermal stability (up to about 300 °C for PU_3 and PU_31 and up to 190 °C for PU_3T2 and PU_31T2). Even if the combustion process of the rocket propellants begins at lower temperatures than the neat polyurethane binders, they still can be safely utilized, each composite possessing decomposition onset temperatures above 165 °C.

DMA profile of the polyurethanes from recycled PET plasticized with TEGDN displayed a very low glass transition temperature (−53 °C), while for the propellant based on it, the T_g_ was slightly higher due to the presence of solid loading. DMA analysis also demonstrated that the polyurethanes and propellant specimens based on polyester-polyols synthesized from PET waste possess a good capacity for absorbing and dissipating energy. Tensile and compressive test results also showed that the developed specimens (polyurethanes and propellants) have an acceptable mechanical behavior, in accordance with the minimal requirements for solid rocket propellant binders. Thus, in comparison with the existing HTPB binders present in available rocket propellants, despite their poorer mechanical resistance (which is situated within some tolerable values), the binders based on recycled PET waste can enhance the thermal performances of the rocket propellant and contribute to a less polluted environment. Additionally, the higher flexibility will ensure resilience, a superior shock resistance necessary for the prevention of crack formation and adhesion failure, thus insuring the geometric stability of the propellant grain.

Small-scale real firing testing of the novel solid composite propellants indicated ballistic performances in accordance with those exhibited by state-of-the-art solid propellants (adequate values for pressure and combustion rate, improved energetic performances). We can conclude that the extensive study herein reported offers a comprehensive image of the possibility to replace the existing binders, such as HTPB, with binders derived from PET, while maintaining high performance standards, thus substantially minimizing the environmental impact of the rocket propellants. This ecological approach could be integrated into the life cycle assessment of environmentally responsible rocket propellants that should be developed in the future. By developing future “greener” rocket propellants based on polyols synthesized from PET waste and “clean” oxidizers, a great contribution could be added to the circular economy process and to the global effort to protect the environment and human health.

## Figures and Tables

**Table 1:** The composition of the investigated polyols.

Sample	Composition	-OH^SET^/-OH^RP^ Ratio ^1^	TEGDN (wt%) ^2^
S50	SET	-	-
SRP1	SET:RP1	0.75:0.25	-
SRP1T1	SET:RP1 + TEGDN	0.75:0.25	15%
SRP1T2	SET:RP1 + TEGDN	0.75:0.25	30%
SRP1	SET:RP2	0.75:0.25	-
SRP1T1	SET:RP2 + TEGDN	0.75:0.25	15%
SRP1T2	SET:RP2 + TEGDN	0.75:0.25	30%

^1^ -OH molar ratios for SET and RP blends. ^2^ TEGDN added to SET:RP blends, as weight percentage calculated from the total weight of the binder.

**Table 2:** Composition of the polyurethane binders.

Sample	Composition	-NCO/-OH Ratio	TEGDN (wt%)
PU_1	DES:SET	1:1	-
PU_2		3:2	-
PU_3		2:1	-
PU_1T1	DES:SET+TEGDN	1:1	15%
PU_2T1		3:2	15%
PU_3T1		2:1	15%
PU_1T2		1:1	30%
PU_2T2		3:2	30%
PU_3T2		2:1	30%
PU_11	DES:SET: RP1	1:0.75:0.25	-
PU_21		3:1.5:0.5	-
PU_31		2:0.75:0.25	-
PU_11T1	DES:SET:RP1+TEGDN	1:0.75:0.25	15%
PU_21T1		3:1.5:0.5	15%
PU_31T1		2:0.75:0.25	15%
PU_11T2		1:0.75:0.25	30%
PU_21T2		3:1.5:0.5	30%
PU_31T2		2:0.75:0.25	30%
PU_12	DES:SET:RP2	1:0.75:0.25	-
PU_22		3:1.5:0.5	-
PU_32		2:0.75:0.25	-
PU_12T1	DES:SET:RP2+TEGDN	1:0.75:0.25	15%
PU_22T1		3:1.5:0.5	15%
PU_32T1		2:0.75:0.25	15%
PU_12T2		1:0.75:0.25	30%
PU_22T2		3:1.5:0.5	30%
PU_32T2		2:0.75:0.25	30%

**Table 3:** Formulations for the rocket propellant composite.

Sample	Components [wt.%]					
	PU_3	PU_3T2	PU_31	PU_31T2	Al <5 μm	Al 100 μm
ECP_A5	15				12	
ECP_A100	15					12
ECP_B5		15			12	
ECP_B100		15				12
ECP_C5			15		12	
ECP_C100			15			12
ECP_D5				15	12	
ECP_D100				15		12

**Table 4:** Assessment of I_OH_ and I_A_ for the polyester-polyols that resulted from the degradation of PET, through titration methods.

Sample	Properties			
	I_-OH_ mgKOH/g	I_A_ mgKOH/g	M_n,_ calc. *	Description
RP1	30.86	1.47	3470	Pale yellow, viscous liquid
RP2	33.36	1.08	3257	Light brown, viscous liquid

* Mn calc. = (2 × 56.1 × 1000)/(I_A_ + I_OH_) [41,42].

**Table 5:** Tensile characteristics of polyurethane films.

Sample	Young’s Modulus (MPa)	Maximum Tensile Stress (MPa)	Maximum Tensile Strain	Deformation Energy (Tensile Toughness) (J m^−3^)
PU_3	18.01 ± 2.06	10.19 ± 1.54	0.56 ± 0.05	3.95 ± 0.82
PU_31	13.27 ± 1.86	9.22 ± 1.29	0.69 ± 0.07	3.16 ± 0.66
PU_3T2	12.79 ± 0.76	3.30 ± 0.31	0.26 ± 0.02	0.45 ± 0.08
PU_31T2	5.92 ± 1.78	1.97 ± 0.39	0.34 ± 0.03	0.32 ± 0.06

**Table 6:** Tensile properties for the novel composite rocket propellants.

Sample	Young’s Modulus (MPa)	Tensile Stress (MPa)	Tensile Strain (%)
ECP_B5	140.6 ± 0.10	1.3 ± 0.11	0.01
ECP_C5	106.3 ± 0.21	1.1 ± 0.20	0.01
ECP_D5	44.9 ± 0.06	0.4 ± 0.07	0.01

**Table 7:** Compressive strain–stress values of the composite rocket propellants.

Sample	Compressive Strain (%)	Compressive Stress (MPa)
ECP_A5	0.22	0.90 ± 0.13
ECP_A100	0.23	0.52 ± 0.03
ECP_B5	0.22	0.75 ± 0.03
ECP_B100	0.26	0.41 ± 0.01
ECP_C5	0.29	0.49 ± 0.09
ECP_C100	0.23	0.66 ± 0.07
ECP_D5	0.22	0.61 ± 0.04
ECP_D100	0.25	0.38 ± 0.03

## Supplementary Materials

The following are available online at https://www.mdpi.com/article/10.3390/polym13213828/s1. Included are NMR spectra of SRP1, SRP2, SRP1T2, and SRP2T2 (Figures S1–S4), FT-IR of polyurethanes based on RP2 (Figure S5), images of polyurethane binders and curing time (Figure S6), SEM images of polyurethane formulations based on RP1 and RP2 (with and without TEGDN) (Figure S7), EDX mapping of the polyurethane formulations from S7 (Figure S8), tensile stress–strain plots for PU formulations (Figure S9), tensile stress–strain plots for composite propellant formulations (Figure S10), compression test plots for composite formulations (Figure S11), SEM-EDX images of composite propellant formulations (Figure S12), EDX spectra for oxidizer-free and binder-free formulations (Figure S14 and S15), stand burner equipped with subscale rocket motor TRM-35 (Figure S16), structural configuration of ECP_D5 (Figure S17), pyrotechnic composition for propellant ignition (Figure S18), flame configuration of ECP_D5 during combustion (Figure S19), behavior of PUs during manual bending (Figure S20), structural configurations of composite propellants for mechanical analysis (Figure S21), weight and atomic composition of free oxidizer composite formulations (Table S1), weight and atomic composition of free binder solid mixture (Table S2), thermal characteristics for polyurethane films, composite propellant formulations, and energetic plasticizer (Table S3), thermal properties of synthesized polyurethanes (Table S4), the decomposition process of the polyurethanes (weight loss versus temperature) (Table S5), and heat of combustion, specific volume and Tg for our new composite propellants in comparison with the existing commercial formulations (Table S6).
