# Supplementary material for: Novel Polyurethanes Based on Recycled Polyethylene Terephthalate: Synthesis, Characterization, and Formulation of Binders for Environmentally Responsible Rocket Propellants
Source: Polymers (Basel). 2021 Nov 5;13(21):3828. doi: 10.3390/polym13213828 (PMC8587294; doi:10.3390/polym13213828)
Supplement: Supplementary file 1 [file polymers-13-03828-s001.zip › polymers-1423272-supplementary.pdf]

Supplementary Material

Novel Polyurethanes Based on Recycled Polyethylene Terephthalate: Synthesis, Characterization, and Formulation of Binders for Environmentally Responsible Rocket Propellants

Florin-Marian Dîrloman,[1, ‡], Gabriela Toader[1, ‡], Traian Rotariu[1,*], Tudor-Viorel Țigănescu [1,2], Raluca Ginghina [3], Răzvan Petre[3], Florentina Alexe[3], Mihai-Ionuț Ungureanu[1], Edina Rusen[4], Aurel Diacon[4], Adi Ghebaur [4], Monica Duldner[5], Alina Elena Coman[5] and Robert Țincu[6]

1 Military Technical Academy "Ferdinand I", 39-49 George Cosbuc Boulevard, Bucharest, 050141, Romania

2 Military Equipment and Technologies Research Agency, 16 Aeroportului Street, Clinceni, Ilfov, 077025, Romania

3 Research and Innovation Center for CBRN Defense and Ecology, 225 Soseaua Oltenitei, Bucharest, 041327, Romania

4 Faculty of Applied Chemistry and Materials Science, University Politehnica of Bucharest, 1-7 Gh. Polizu Street, Bucharest, 011061, Romania

5 National Institute of Research and Development for Chemistry and Petrochemistry, 202 Splaiul Independentei, Bucharest, 060041, Romania

6 Center for Organic Chemistry "C.D. Nenitescu" of the Romanian Academy, 202B Splaiul Independentei, Bucharest, 060023, Romania

‡ co-first authors, equally contributed to this work

*Correspondence: author: traian.rotariu@gmail.com

Table of Contents

Figure S1 – 1H- NMR spectra for SRP1 3
Figure S2 – 1H- NMR spectra for SRP2 3
Figure S3 – 1H- NMR spectra for SRP1T2 3
Figure S4 – 1H- NMR spectra for SRP2T2 4
Figure S5 – FT-IR spectra of RP2 polyurethane compounds 4
Figure S6 – Binder characterization regarding aspect and time reaction 5
Figure S7 – SEM images of polyurethanes formulations based on RP1 and RP2 for 3:2 and 2:1 ratio with and without TEGDN 6
Figure S8 EDX mapping of the polyurethane formulations 6
Figure S9 – Tensile charts for PU formulations 7
Figure S10 – Tensile charts for composite propellant formulations 8
Figure S11 – Compressive test charts for composite formulations 9
Figure S12 – SEM-EDX images of composite propellant formulations 11
Figure S13 – EDX spectra for free oxidizer composite formulations 11
Figure S14 – SEM-EDX images of free binder solid mixture 12
Figure S15 – EDX spectra for free binder solid mixture 12
Figure S16 – Stand burner equipped with Subscale Rocket Motor TRM-35 13
Figure S17 – Structural configuration of ECP_D5 13
Figure S18 – Pyrotechnic composition for propellant ignition 13
Figure S19 – Flame configuration of ECP_D5 during the combustion 14
Figure S20 – Behavior of PUs during manual bending 14
Figure S21 – Structural configurations of composite propellants for mechanical analysis 15
Table S1. Weight and atomic composition of free oxidizer composite formulations 15
Table S2. Weight and atomic composition of free binder solid mixture 15
Table S3. Thermal characteristics for polyurethane films, composite propellant formulations, and energetic plasticizer 15
Table S4. Thermal properties of synthesized polyurethanes 15
Table S5. The decomposition process of the polyurethanes (weight loss versus temperature) 16
Table S6. Heat of combustion, specific volume and Tg for our new composite propellants in comparison with the existing commercial formulations 16

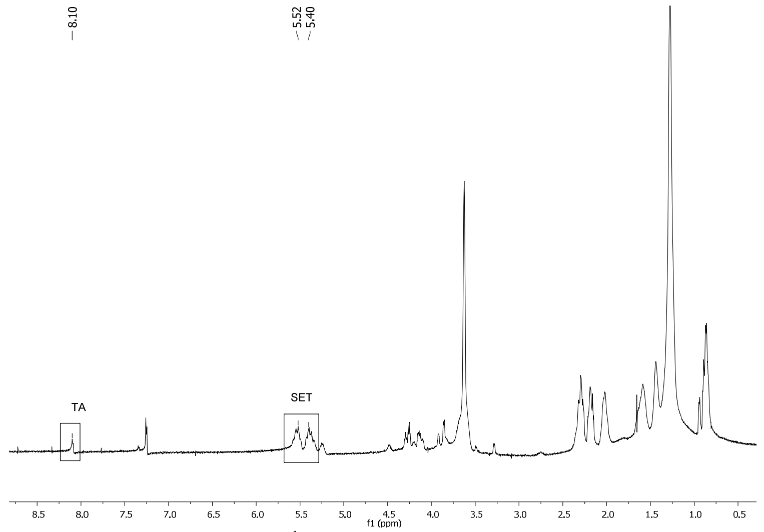

Figure S1. – ^{1}H- NMR spectra for SRP1.

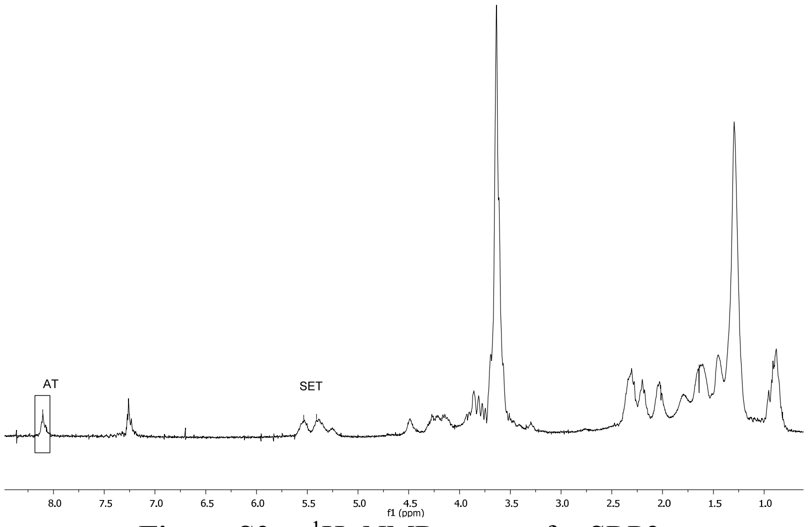

Figure S2. – ^{1}H- NMR spectra for SRP2.

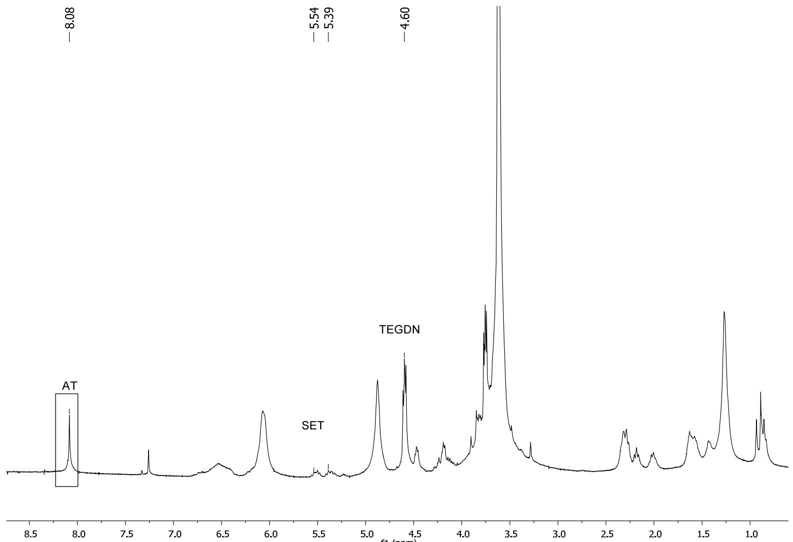

Figure S3. – ^{1}H- NMR spectra for SRP1T2.

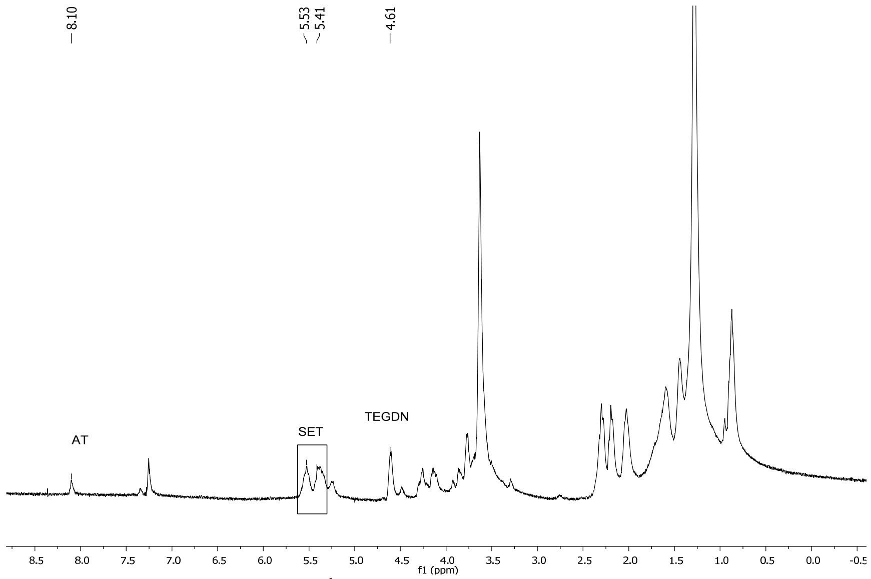

Figure S4. – ¹H- NMR spectra for SRP2T2.

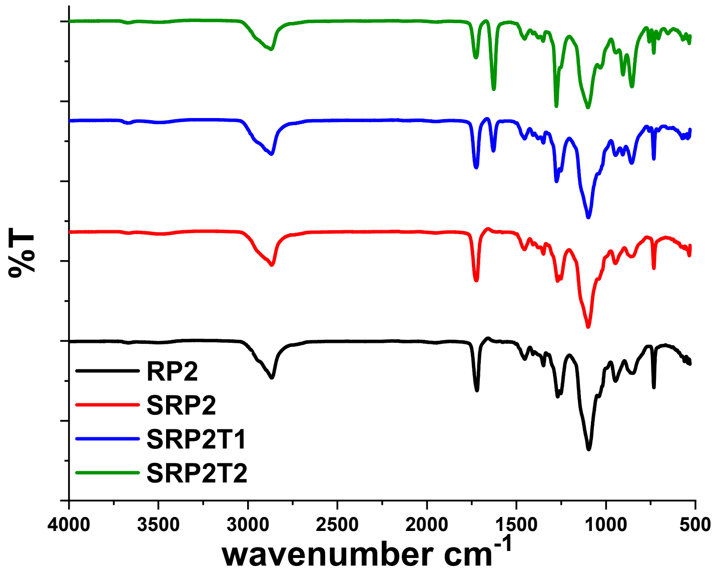

Figure S5. – FT-IR spectra of polyurethanes based on RP2.

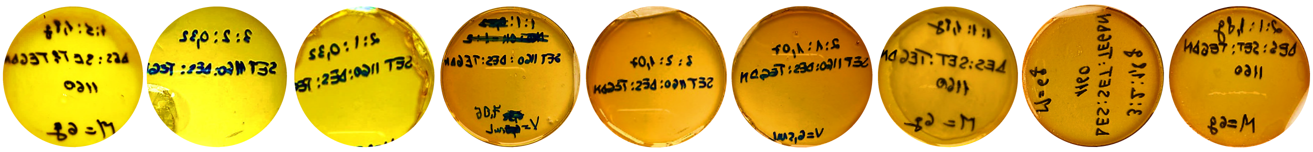

PU_1	PU_2	PU_3	PU_1T1	PU_2T1	PU_3T1	PU_1T2	PU_2T2	PU_3T2
Flexible, bubble-free film, compact structure Tc=72 h	Flexible film, few bubbles, compact structure Tc=44 h	Not so flexible, few bubbles, compact structure Tc=18 h	Flexible, bubble-free film, compact structure Tc=94 h	Flexible, bubble-free film, compact structure Tc=60 h	Flexible, bubble-free film, compact structure Tc=24 h	Flexible, bubble-free film, compact structure Tc=128 h	Flexible, bubble-free film, compact structure Tc=102 h	Flexible, bubble-free film, compact structure Tc=50 h

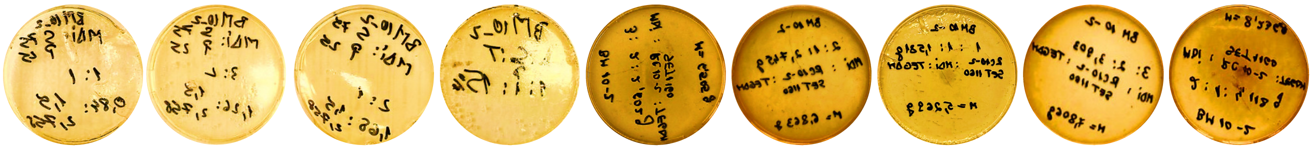

PU_11	PU_21	PU_31	PU_11T1	PU_21T1	PU_31T1	PU_11T2	PU_21T2	PU_31T2
Sticky, gelatinous, bubble-free film Tc=86 h	Flexible film, few bubbles,, compact structure Tc=52 h	Flexible film, few bubbles,, compact structure Tc=32 h	Incomplete reaction Tc=n/a h	Flexible, bubble-free film, compact structure Tc=102 h	Flexible, bubble-free film, compact structure Tc=58 h	Incomplete reaction Tc=n/a h	Flexible, bubble-free film, compact structure Tc=132 h	Flexible, bubble-free film, compact structure Tc=96 h

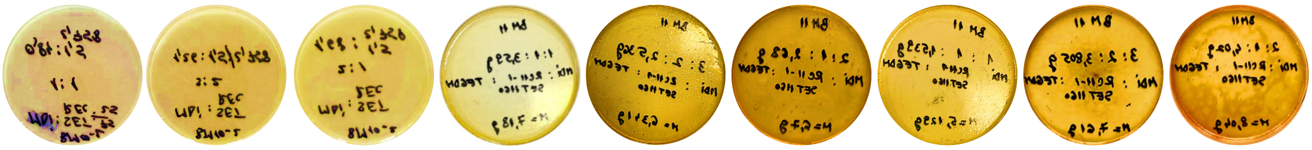

PU_12	PU_22	PU_32	PU_12T1	PU_22T1	PU_32T1	PU_12T2	PU_22T2	PU_32T2
Sticky, gelatinous, bubble-free film Tc=92 h	Flexible film, few bubbles, compact structure Tc=62 h	Flexible film, few bubbles, compact structure Tc=38 h	Incomplete reaction Tc=n/a h	Flexible, bubble-free film, compact structure Tc=102 h	Flexible, bubble-free film, compact structure Tc=64 h	Incomplete reaction Tc=n/a h	Flexible, bubble-free film, irregular surface Tc=142 h	Flexible, bubble-free film, irregular surface Tc=102 h

Figure S6. – Brief table comprising the aspect, properties, and the time of curing for each polyurethane binder.

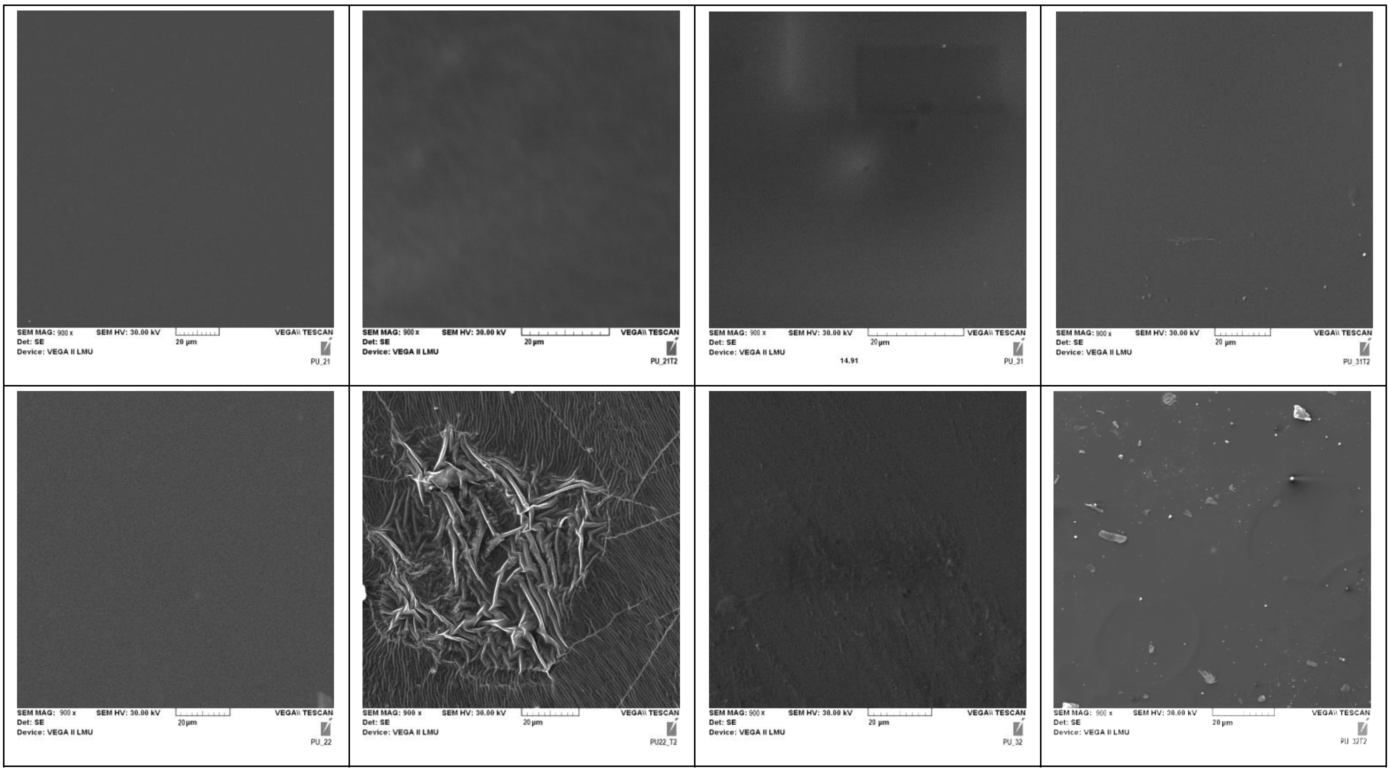

Figure S7. – SEM images of polyurethanes formulations based on RP1 and RP2 (with and without TEGDN).

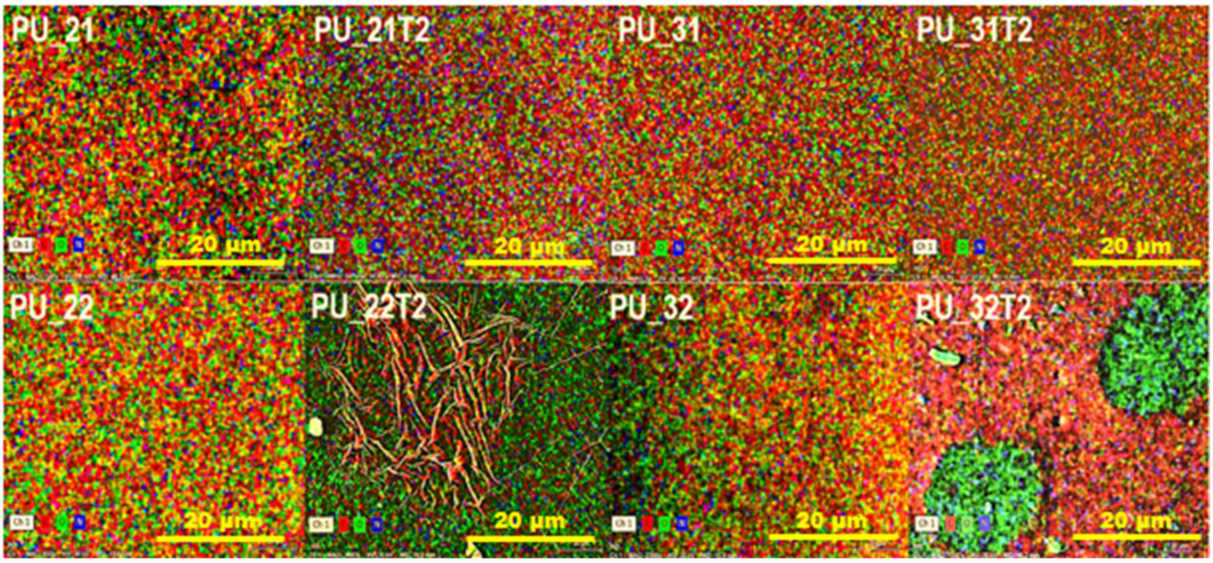

Figure S8. EDX mapping of the polyurethane formulations from S7.

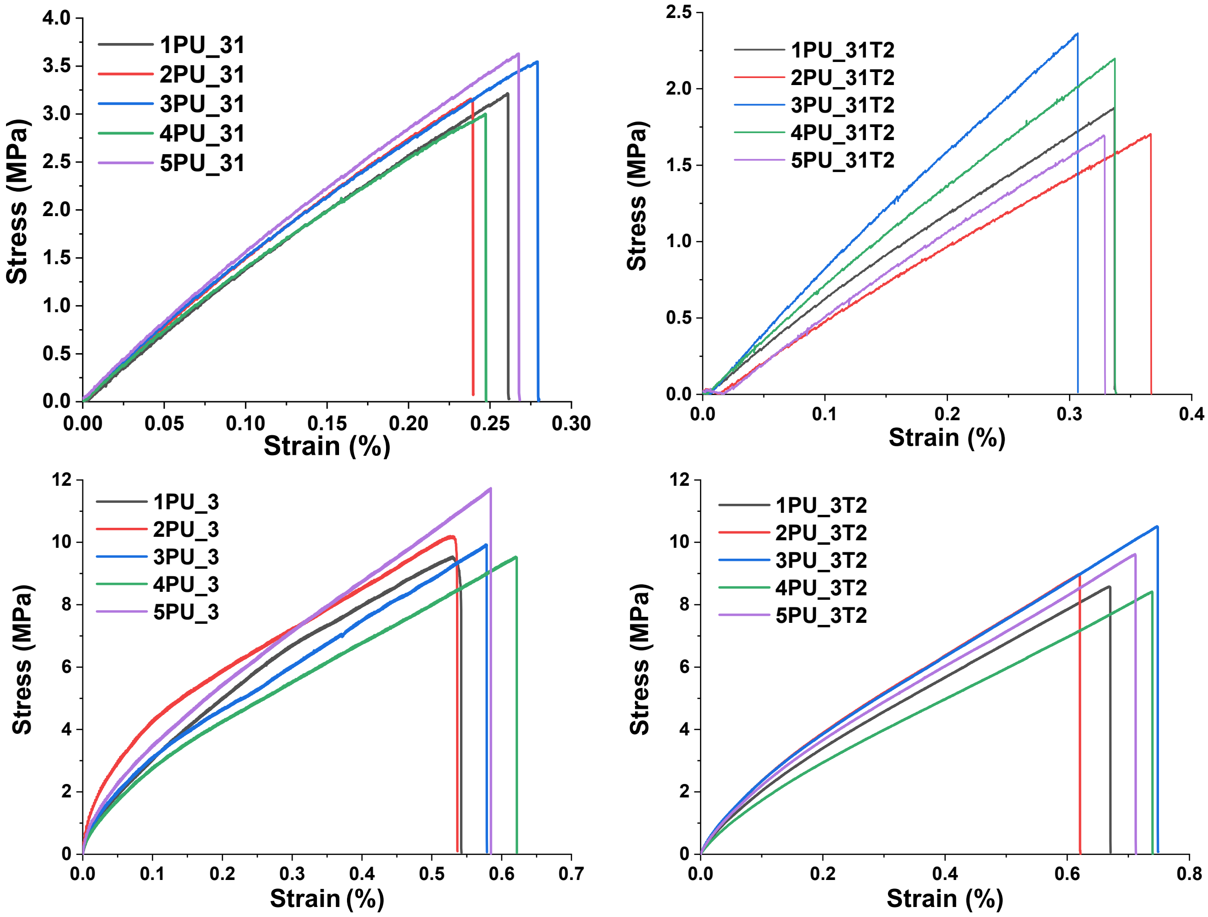

Figure S9. – Tensile stress-strain plots for PU formulations.

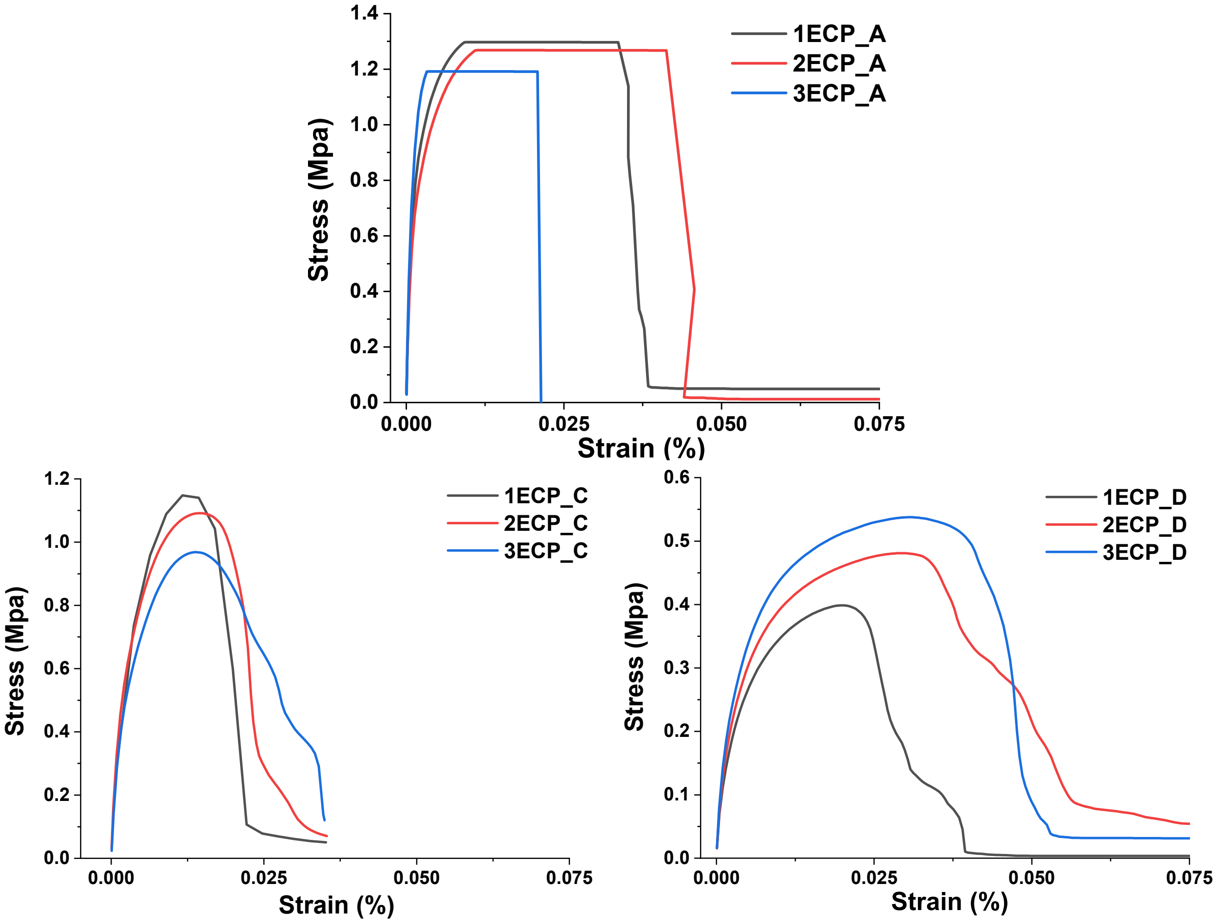

Figure S10. – Tensile stress-strain plots for composite propellant formulations.

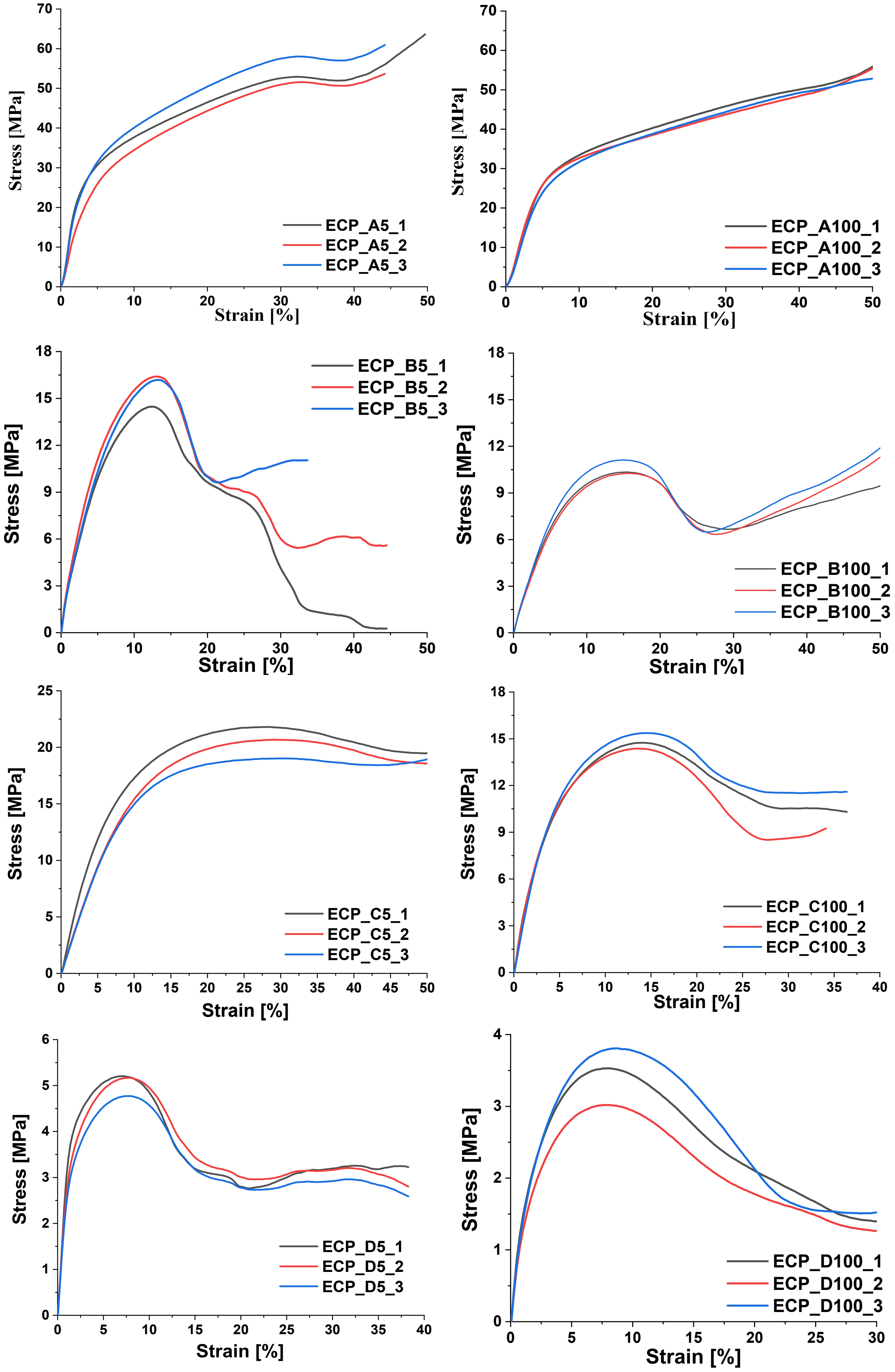

Figure S11. – Compression tests plots for composite formulations.

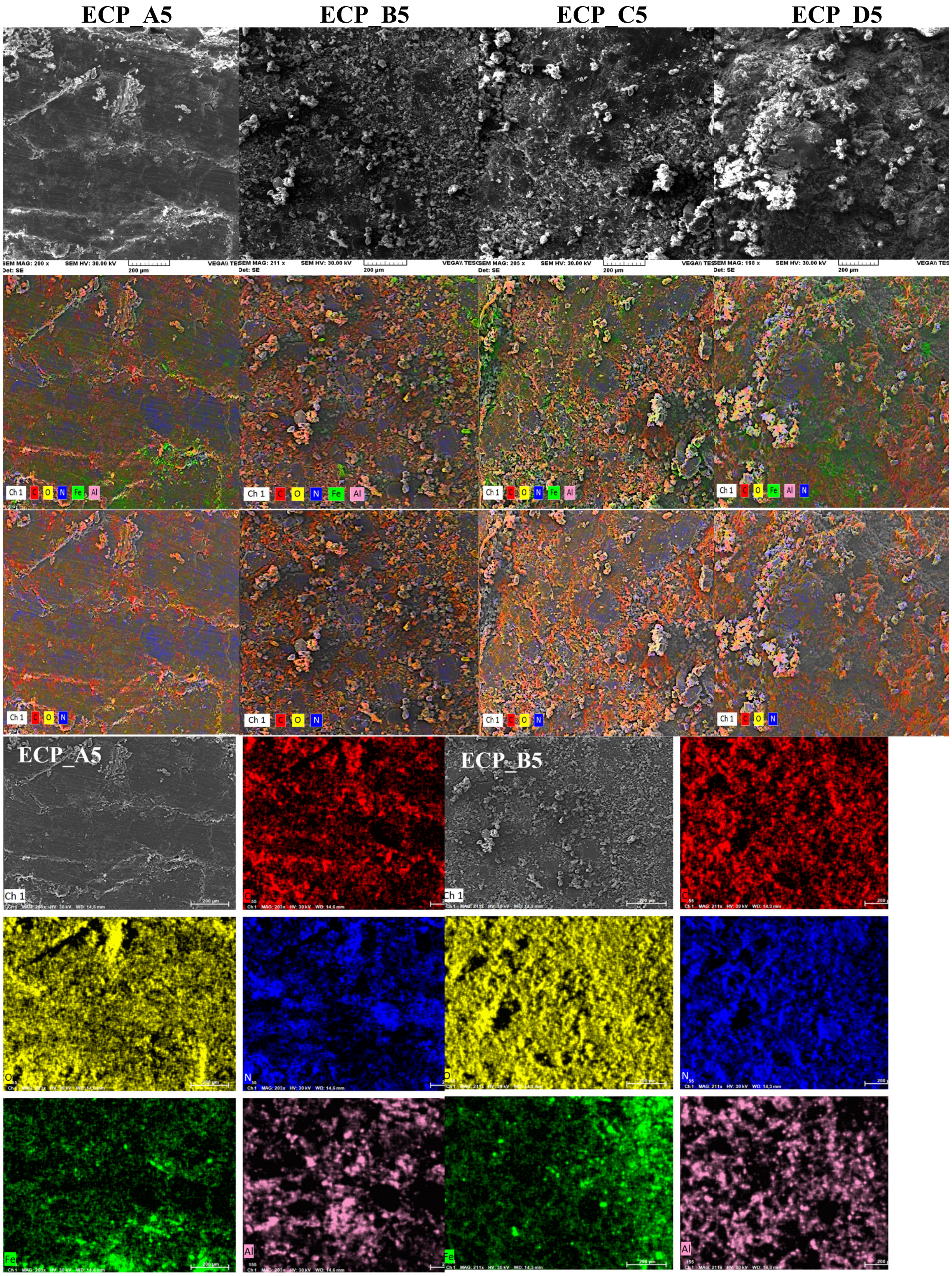
ECP_A5
ECP_B5
ECP_C5
ECP_D5
SEM MAG: 200 x SEM HV: 30.00 kV Det: SE 200 μm VEGA\\ TES
SEM MAG: 211 x SEM HV: 30.00 kV Det: SE 200 μm VEGA\\ TES
SEM MAG: 205 x SEM HV: 30.00 kV Det: SE 200 μm VEGA\\ TES
SEM MAG: 198 x SEM HV: 30.00 kV Det: SE 200 μm VEGA\\ TES
Ch 1 C O N Fe Al
Ch 1 C O N Fe Al
Ch 1 C O N Fe Al
Ch 1 C O Fe Al N
Ch 1 C O N
Ch 1 C O N
Ch 1 C O N
Ch 1 C O N
ECP_A5
ECP_B5
Ch 1
C
O
N
Fe
Al

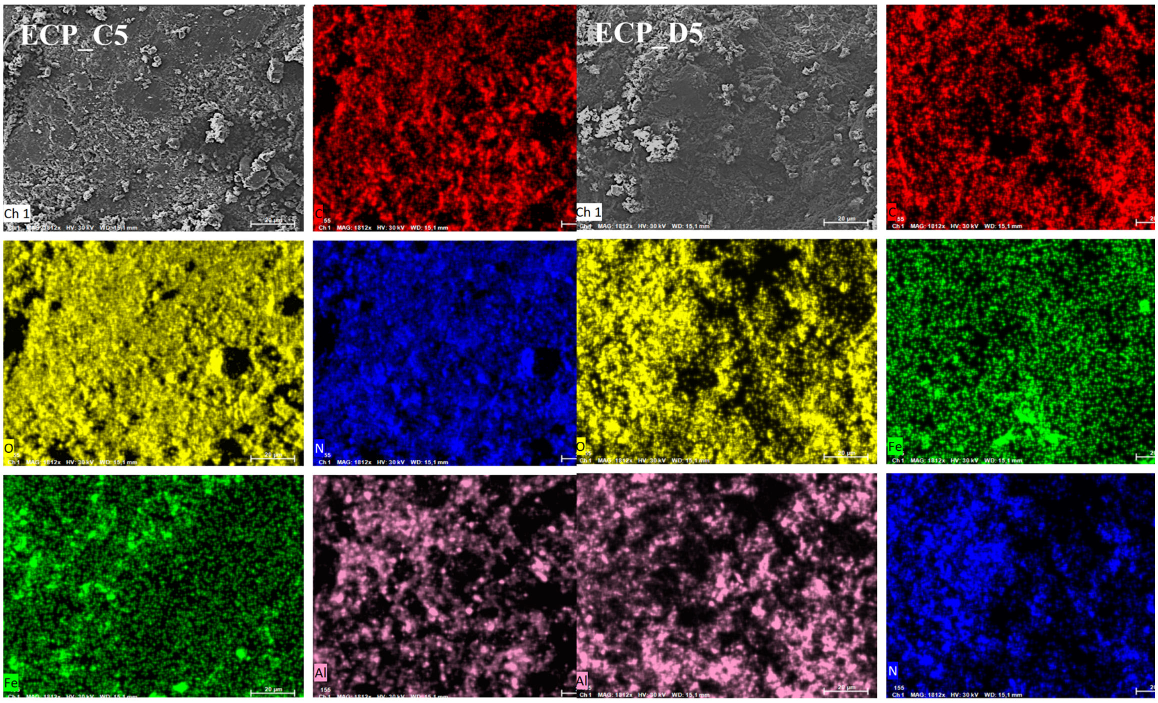

Figure S12. – SEM-EDX images of composite propellant formulations.

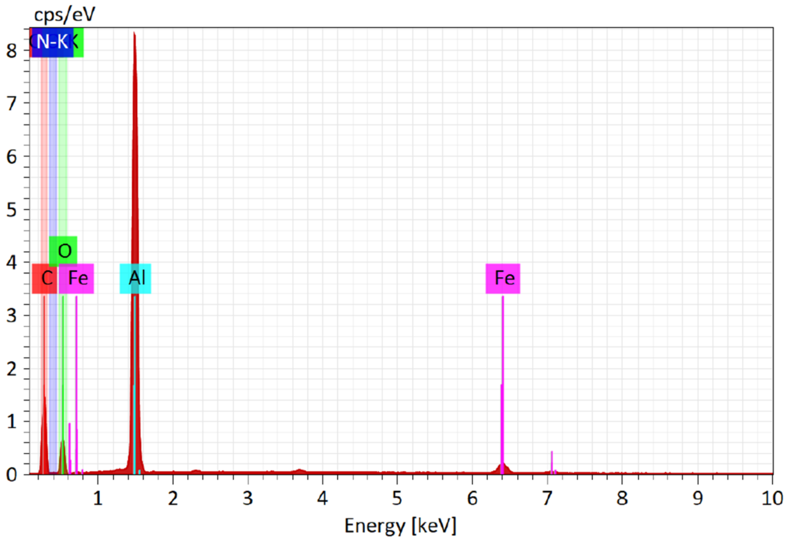

Figure S13. – EDX spectra for oxidizer-free composite formulations .

Table S1. Weight and atomic composition of oxidizer-free composite formulations.

Element	At. No.	Netto	Mass [%]	Mass Norm. [%]	Atom [%]	abs. error [%] (1 sigma)	rel. error [%] (1 sigma)
Carbon	6	52579	119,49	54,32	65,62	6,53	5,47
Oxygen	8	25392	47,09	21,41	19,42	2,59	5,50
Aluminium	13	426660	41,21	18,74	10,08	1,74	4,21
Nitrogen	7	1755	9,77	4,44	4,60	0,59	6,00
Iron	26	20749	2,39	1,09	0,28	0,05	2,18
		**Sum**	**219,96**	**100,00**	**100,00**		

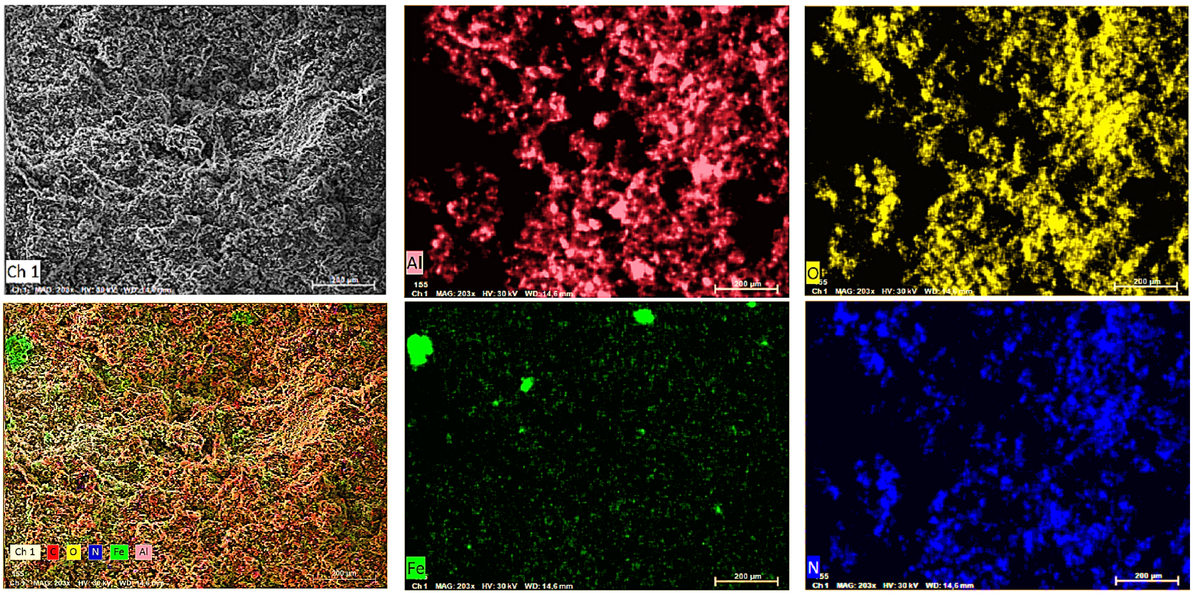

Figure S14. – SEM-EDX images of binder-free solid mixture.

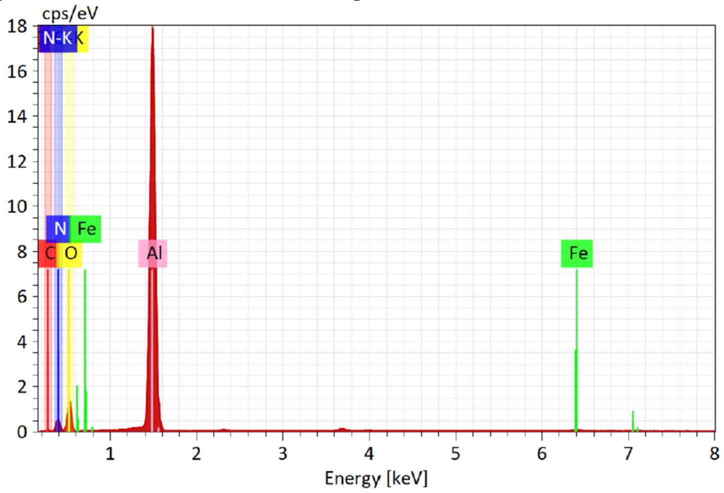

Figure S15 – EDX spectra for binder-free solid mixture.

Table S2. Weight and atomic composition of free binder solid mixture.

Element	At. No.	Netto	Mass [%]	Mass Norm. [%]	Atom [%]	abs. error [%] (1 sigma)	rel. error [%] (1 sigma)
Aluminium	13	917245	58,74	40,03	28,82	2,30	3,91
Oxygen	8	52068	56,50	38,58	43,88	2,57	5,42
Nitrogen	7	21162	30,96	21,10	27,21	1,69	5,47
Carbon	6	0	0,00	0,00	0,00	0,00	4,36
Iron	26	5482	0,54	0,29	0,09	0,01	3,09
		**Sum**	**146,75**	**100,00**	**100,00**		

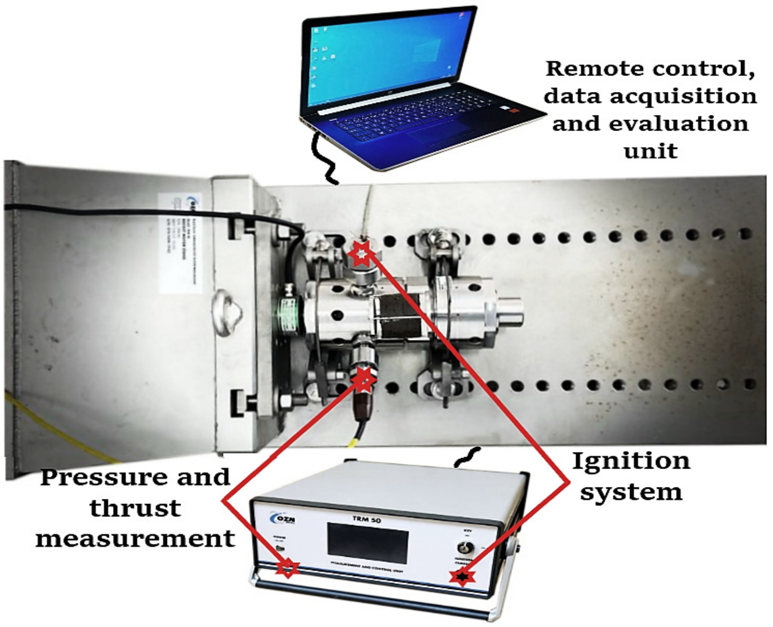

Figure S16. – Stand burner equipped with Subscale Rocket Motor TRM-35.

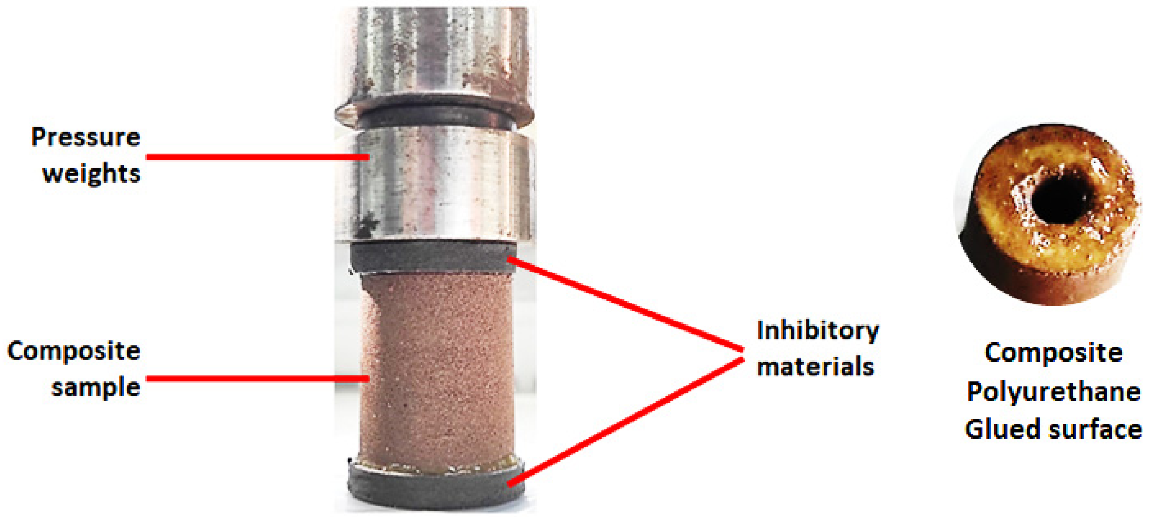

Figure S17. – Structural configuration of ECP_D5.

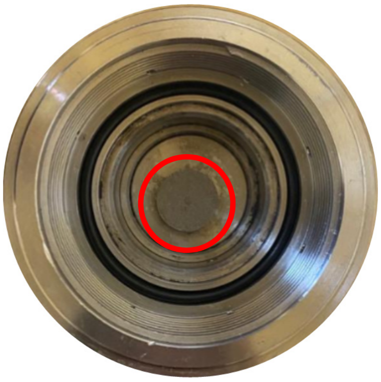

Figure S18. – Pyrotechnic composition for propellant ignition.

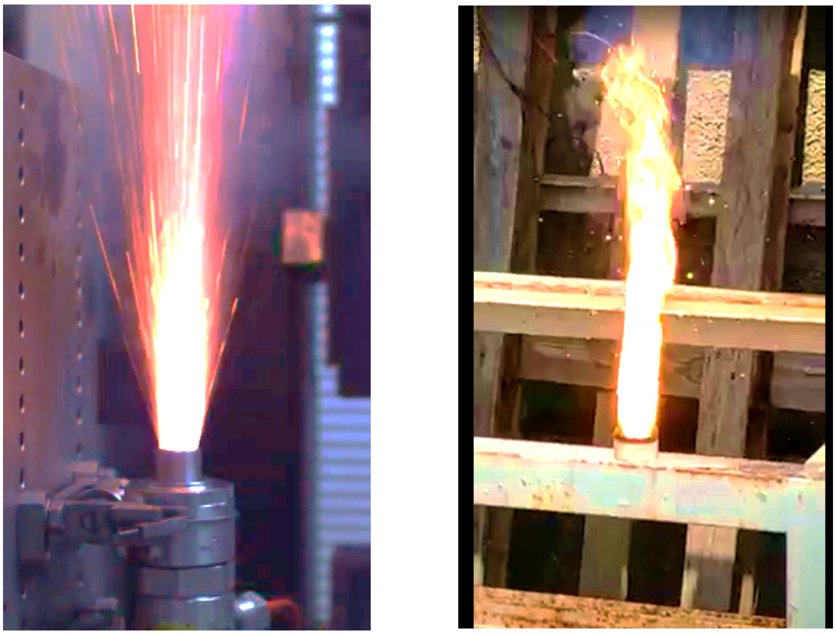

a. b.

Figure S19. – Flame configuration of ECP_D5 during the combustion (**a.** at nozzle exit; **b.** outdoor).

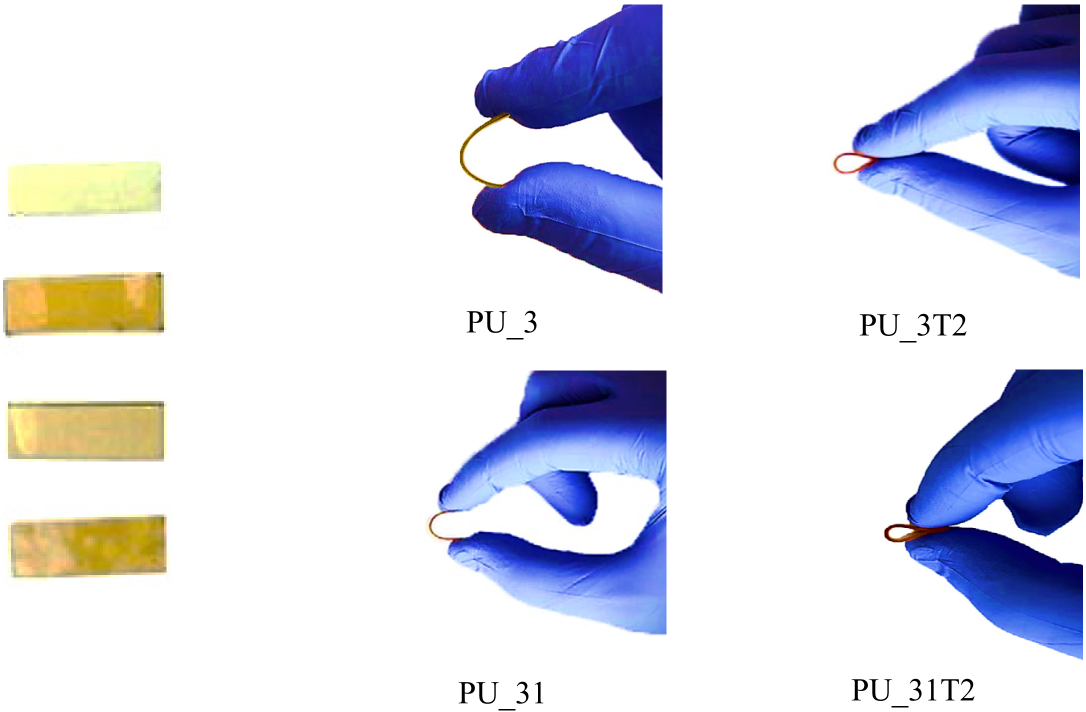

Figure S20. – Behavior of PUs during manual bending.

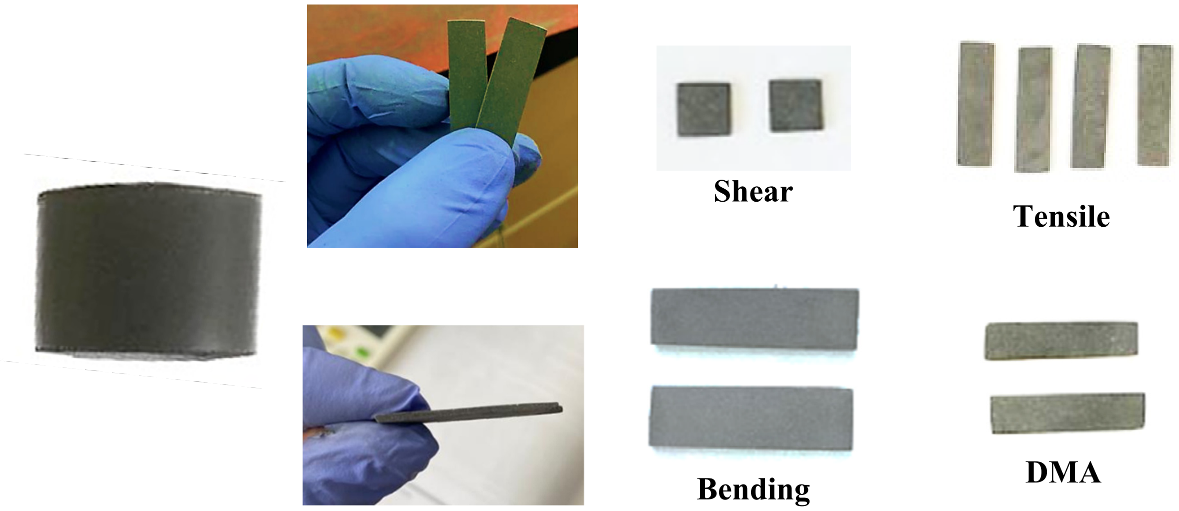

Figure S21. – Structural configurations of composite propellants for mechanical analysis.

Table S3. Thermal characteristics for polyurethane films, composite propellant formulations, and energetic plasticizer.

Sample	Evaluation results [°C]					
	Beginning temperature	Onset	Top	Max. Difference	End Temperature	Peak Type
**PU_2**	208.6	231.7	263.7	0.6	283.8	Exotherm
**TEGDN**	149.4	191.8	193.7	44.6	225.7	Exotherm
**PU_2T1**	161.5	179.2	200.8	3.9	228.7	Exotherm
**PU_2T2**	154.0	185.2	196.6	7.8	228.3	Exotherm
**PU_21T1**	148.3	182.3	199.4	6.2	200.2	Exotherm
**PU_21T2**	142.2	193.7	194.9	40.3	207.7	Exotherm
**PU_3**	208.8	239.5	278.7	0.7	289.7	Exotherm
**PU_3T1**	152.4	179.8	198.6	4.7	233.6	Exotherm
**PU_3T2**	143.1	185.6	196.2	14.4	227.9	Exotherm
**PU_31T1**	159.1	180.7	197.4	6.5	229.9	Exotherm
**PU_31T2**	150.9	190.3	191.2	52.4	202.5	Exotherm
**ECP_A5**	160.5	164.1	54.1	2.5	299.9	Exotherm
**ECP_B5**	155.8	165.3	183.7	32.8	195.8	Exotherm
**ECP_D5**	159.5	176.5	183.8	75.1	194.3	Exotherm

Table S4. Thermal properties of synthesized polyurethanes.

Sample	$T_{10\%}$[1] [°C]	T_{max1}[2] [°C]	T_{max2}[2] [°C]	T_{max3}[2] [°C]	T_{max4}[2] [°C]
**PU_3**	306.1	313.6	371.1	456.1	-
**PU_31**	313.5	313.5	410.9	-	-
**PU_3T2**	197.6	190.1	315.1	370.1	457.6
**PU_31T2**	181.4	193.9	326.4	408.9	-

[1]decomposition onset temperature (measured at 10 % weight loss).
[2]the maximum decomposition temperatures, corresponding to the maximum of DTG peaks.

Table S5. The decomposition process of the polyurethanes (weight loss versus temperature).

Sample	Weight loss [%]								
	200°C	250°C	300°C	350°C	400°C	450°C	500°C	550°C	600°C
**PU_3**	0.5	0.8	7.1	32.1	60.3	70.3	92.9	94.2	95.1
**PU_31**	1.1	1.4	6.4	19.4	43.4	77.4	85.9	87.8	88.6
**PU_3T2**	10.1	13.8	20.2	37.9	55.9	73.1	91.9	94.3	95.2
**PU_31T2**	18.4	25.1	29.5	39.7	58.8	81.7	87.5	88.9	89.7

Table S6. Heat of combustion, specific volume and T_g for our new composite propellants in comparison with the existing commercial formulations.

Sample	Qv [cal/g]	Vsp [l/kg]	T_g
**HTPB composite propellant**	1700	507	-72°C
**Double based propellants**	1020	798	-35.5°C
**ECP_D5**	1002	693	-32°C
**ECP_B5**	1010	658	-61°C
